# Disease dynamics and costly punishment can foster socially imposed monogamy

**DOI:** 10.1038/ncomms11219

**Published:** 2016-04-12

**Authors:** Chris T. Bauch, Richard McElreath

**Affiliations:** 1 Department of Applied Mathematics, University of Waterloo, 200 University Avenue West, Waterloo, Ontario, Canada N2L 3G1; 2 Department of Human Behavior, Ecology, and Culture, Max Planck Institute for Evolutionary Anthropology, Deutscher Platz 6, 04103 Leipzig, Germany

## Abstract

Socially imposed monogamy in humans is an evolutionary puzzle because it requires costly punishment by those who impose the norm. Moreover, most societies were—and are—polygynous; yet many larger human societies transitioned from polygyny to socially imposed monogamy beginning with the advent of agriculture and larger residential groups. We use a simulation model to explore how interactions between group size, sexually transmitted infection (STI) dynamics and social norms can explain the timing and emergence of socially imposed monogamy. Polygyny dominates when groups are too small to sustain STIs. However, in larger groups, STIs become endemic (especially in concurrent polygynist networks) and have an impact on fertility, thereby mediating multilevel selection. Punishment of polygynists improves monogamist fitness within groups by reducing their STI exposure, and between groups by enabling punishing monogamist groups to outcompete polygynists. This suggests pathways for the emergence of socially imposed monogamy, and enriches our understanding of costly punishment evolution.

## Abstract (web summary)

Many human societies transitioned from polygyny to socially imposed monogamy as group sizes increased. Using a simulation model, the authors show that sexually transmitted infections impose heavier fitness penalties on polygynists as group size grows, enabling monogamists who punish polygyny to thrive in large groups.

## Graphical abstract

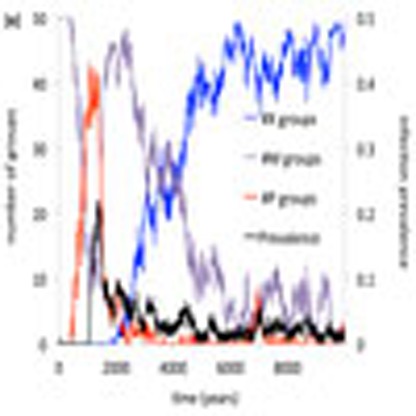

The majority of historically known human societies have allowed, and still allow, polygynous mating^1^,^2^. Yet, in some of the most successful agricultural societies, polygyny was gradually replaced by socially imposed monogamy, beginning with the transition from hunter–gatherers to agriculturalists and the resulting larger residential groups^3^,^4^. Subsequently, a disproportionate amount of the world's population growth in recent millennia has been driven by a relatively small number of societies, many of which practice socially imposed monogamy^5^.

Social monogamy characterizes many animal species, and the role of factors such as infanticide and the spatial distribution of females has been explored in the literature^6^,^7^. However, the social imposition of monogamy via injunctive social norms appears to be particular to humans. This phenomenon is puzzling in two ways. First, from an evolutionary perspective, polygynous males can more readily translate increasing wealth into increasing reproductive success. Therefore, it is unclear why patriarchal societies would voluntarily limit numbers of wives. Second, socially imposed monogamy requires costly punishment by those who impose the norm^8^,^9^. Costly punishment can accrue advantages to the punisher (that is, those who pay a cost to impose a penalty on others exhibiting a certain behaviour) if used to enforce cooperation, for example. However, costly punishment is susceptible to ‘second-order free-riding', whereby non-punishers can reap the benefits created by punishers, without having to pay the costs of punishing^10^. The origin of socially imposed monogamy represents an important knowledge gap because sexual behaviour is highly regulated in many human societies^11^.

Previous hypotheses for the transition from polygyny to socially imposed monogamy have invoked female choice, male power dynamics, technological impacts, cultural group selection and pathogen stress^12^,^13^,^14^,^15^,^16^,^17^,^18^. Any of these may be part of the answer. Moreover, human behaviours are complex phenomena often determined by multiple mechanisms. Therefore, we speculate that multiple mechanisms supported the emergence of socially imposed monogamy. However, hypotheses stressing individual benefits face the obstacle of explaining costly social imposition, and hypotheses that stress group benefits must overcome individual incentives against costly punishment. The timing of the emergence of socially imposed monogamy—with the advent of agriculture and larger resident populations—also requires explanation.

We hypothesize that bacterial sexually transmitted infections (STIs) fostered the emergence of socially imposed monogamy, by making costly punishment of non-monogamists advantageous to punishers at individual and group levels of selection. STIs can impose enormous selective pressures on humans^19^,^20^,^21^. In the absence of modern interventions such as antibiotics, latex condoms or contact tracing, bacterial STIs such as syphilis, chlamydia and gonorrhea can cause very high rates of infertility and thus major demographic impacts^19^,^20^,^22^,^23^,^24^. Therefore, we speculate that STI-imposed selective pressures on our ancestors could have been even larger.

Under certain conditions, populations with overlapping sexual partnerships, or ‘concurrency', exhibit higher STI prevalence than populations of serial monogamists (who only have one partner at a time), even when the total number of partnerships is the same^25^,^26^,^27^,^28^. In regions dominated by small and scattered residential groups, a newly introduced STI is more likely to go extinct due to stochastic fadeout^29^; hence, concurrency may not disadvantage polygyny. However, in regions where technologies such as agriculture have facilitated higher population densities and greater connectivity between groups, STIs can more easily become endemic. In this regime, we hypothesize that polygyny becomes disadvantageous relative to monogamy because of concurrency. Thus, under certain epidemiological conditions, monogamy may outperform polygyny under within-group competition. In addition, groups with individuals who enforce monogamous social norms and thus maintain low within-group STI prevalence may outcompete polygynous groups, who suffer reduced population size from STI health burdens. Such interaction between group size, disease dynamics and social norms may have contributed to the association of monogamy with large, integrated agricultural societies.

We emphasize that our hypothesis concerns mating, not marriage *per se.* Some societies, such as the ancient Romans, imposed monogamous marriage but permitted extramarital sexual relations in brothels, so long as moderation was exercised^30^. On the other hand, some partible paternal societies in South America encourage extramarital polyandrous mating^31^. Hence, although marriage and mating are closely related, there is not always one-to-one correspondence.

These examples illustrate the enormous variability in sexual norms^11^, which should be expected if norms are complex phenomena determined by multiple mechanisms. It is therefore improbable that any single hypothesis could explain the entire range of observed behaviours. However, despite this variability, our hypothesis is testable because it concerns an overall trend in behaviour. In populations where our hypothesized mechanism is in operation, we should observe (when controlling for other variables) that the prevalence of polygyny declines with increasing group size on account of polygynists experiencing a higher bacterial STI prevalence that puts them at a fertility disadvantage.

Testing this hypothesis would require data on bacterial STI prevalence in hunter–gatherer and agriculturalist groups before the advent of modern medicine. Such data appear to be scant or non-existent. In the standard cross-cultural sample from Murdock and White's ethnographic atlas^1^, we find that the proportion of polygynous groups decreases with group size (*P*<10^−3^, *r*=−0.67, weighted linear least-squares regression; Supplementary Table 1 and Supplementary Fig. 1). In addition, the frequency distribution of group sizes of polygynous groups is smaller on average, and significantly different, than the frequency distribution of group sizes of monogamous groups (*P*=0.0073 Student's *t*-test; *P*=0.089 two-sample Kolmogorov–Smirnov test). However, the standard cross-cultural sample does not contain data on bacterial STIs. Moreover, this preliminary analysis does not control for confounding variables such as modern disease interventions, cultural phylogeny and other factors that may influence the relationship between polygyny and group size.

An empirical test of the hypothesis is desirable despite these challenges. However, our objective was to validate the basic logic of the hypothesis. This is imperative since the hypothesis invokes complex nonlinear interactions between natural and social dynamics. We present an empirically grounded simulation model based on known demographic parameters of hunter–gatherers and agriculturalists, and established transmissibility and natural history profiles of bacterial STIs. We use the model to investigate the dynamics of sexual norm evolution in small and large residential groups, showing that differential bacterial STI disease burden in polygynists versus monogamists can robustly support the evolution of socially imposed monogamy in larger residential groups, at empirically realistic model parameter values.

## Results

### Baseline small and large group scenarios

We developed an empirically driven agent-based model that captures interactions between social dynamics and disease dynamics^8^,^26^,^32^. Full details appear in the Methods section. Parameter values reflect bacterial STI epidemiology and the known demographics of hunter–gatherers and agriculturalists^33^,^34^,^35^,^36^,^37^,^38^,^39^,^40^,^41^,^42^,^43^,^44^,^45^,^46^,^47^,^48^,^49^,^50^,^51^ (Table 1). We chose bacterial STIs such as gonorrhea, syphilis and chlamydia as our baseline since they have been present in human populations for a relatively long time^52^. Males can play the strategies P (polygynous), M (serial monogamist, non-punisher) or X (serial monogamist, punisher). Single females look for mates with a fixed probability per timestep, sorting available males by an ideal free distribution based on fitness, defined as the current lifetime number of offspring. A proportion of single females practice exogamy, seeking partners outside of their birth group. With a given probability per timestep, a pair can break up, an individual can die or a pair can give birth. Males must help provision the offspring of all of their female partners; hence, the birth probability per pair depends on the number of female partners of the male, although the value of the provisioning parameter was set conservatively to favour polygynists (Table 1). A newly recruited male adopts a strategy through social learning: he randomly selects another male in the group and adopts his strategy if that male has a higher fitness than the group average^53^,^54^. However, a small proportion of males choose a strategy randomly.

As a result of the way partnership dynamics are defined, the sexual interactions in the population can be characterized as a sexual partnership network^25^,^41^,^55^. The network is highly fragmented, consisting of a collection of isolated, slowly evolving pair components and—in the case of polygynous groups—higher-order components with a star topology (triples, quadruples, and so on). The network has a low node degree since partnerships are relatively long lasting, with polygynists typically only having ≤3 partners at a time (≤1 for monogamists) and females only having ≤1 partners. The network has a clustering coefficient of zero, since it is heterosexual, and polygynous groups exhibit a high degree of concurrency^28^,^55^,^56^,^57^,^58^.

At regular time intervals, each individual has a small probability of becoming an index case of a new STI outbreak. Infection spreads from an infected person to a susceptible partner with a certain probability per timestep. A certain proportion of infected persons are rendered infertile, and infected persons die from disease with a certain probability per timestep. The infection has a probability of being cleared in each timestep as well, after which the individual is fully susceptible again. In each timestep, each punishing monogamist pays a fixed cost to incur a penalty on each polygynist in his group (hence, the costs paid/imposed for each individual depend on the frequencies of polygynists/punishing monogamists in the group). Costs and penalties are expressed as a reduced birth rate.

Both the number of groups in the metapopulation and the number of individuals in each group are constrained by their respective carrying capacities. In each timestep, there is a certain probability of two randomly chosen groups competing, with the winner depending partly on group size. Vanquished groups are removed, and half of the victorious group takes over their niche. Initially, all individuals practice polygyny except for a small number of punishing and non-punishing monogamists.

For the large group scenario, the model predicts a population average STI prevalence of 2.9% (Table 2), which is comparable to reported prevalences of gonorrhea, chlamydia and syphilis in the World Health Organization (WHO) African Region^47^ (1.7–4.5% depending on STI and gender).

**Figure 1:**
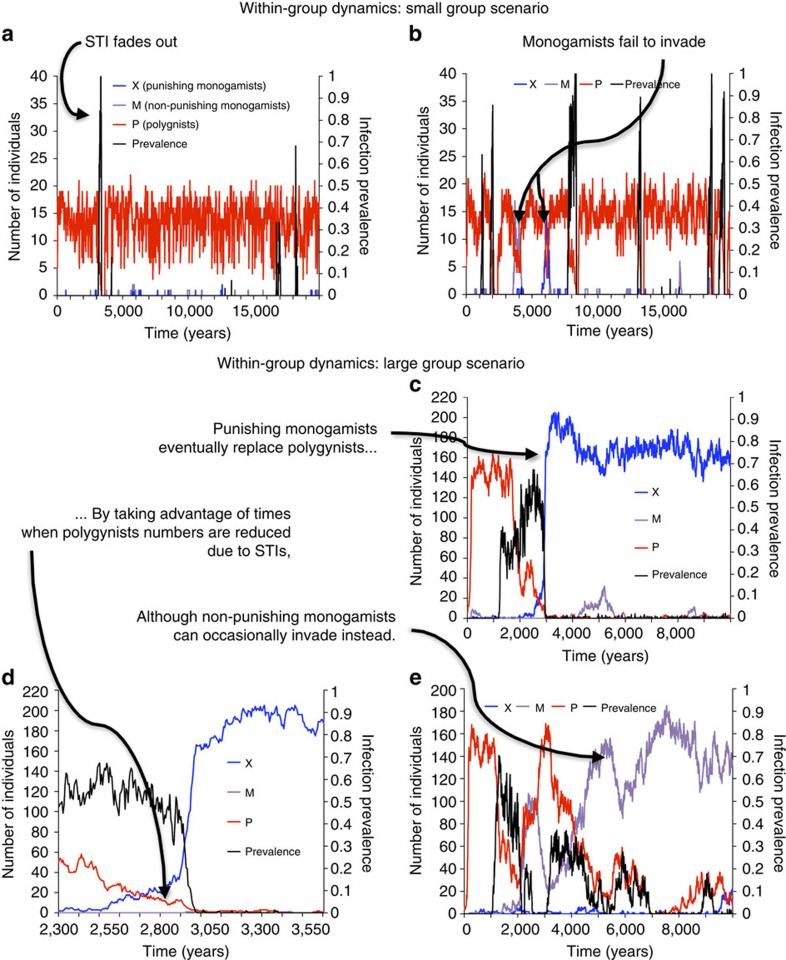
STIs become endemic when groups become large and thereby enable punishing monogamists to take over most groups. Panels show the numbers of punishing monogamists *X* (blue), non-punishing monogamists *M* (purple), polygynists *P* (red) and infection prevalence (proportion infected; black) for the small group scenario in two different groups (**a**,**b**) as well as for the large group scenario in a group over both a long time window (**c**) and a shorter time window showing the transition period (**d**), as well as a different group exhibiting a less common outcome where monogamists dominate (**e**). Group carrying capacity is 30 individuals for the small group scenario (**a**,**b**) and 300 individuals for the large group scenario (**c**–**e**).

**Figure 2 f2:**
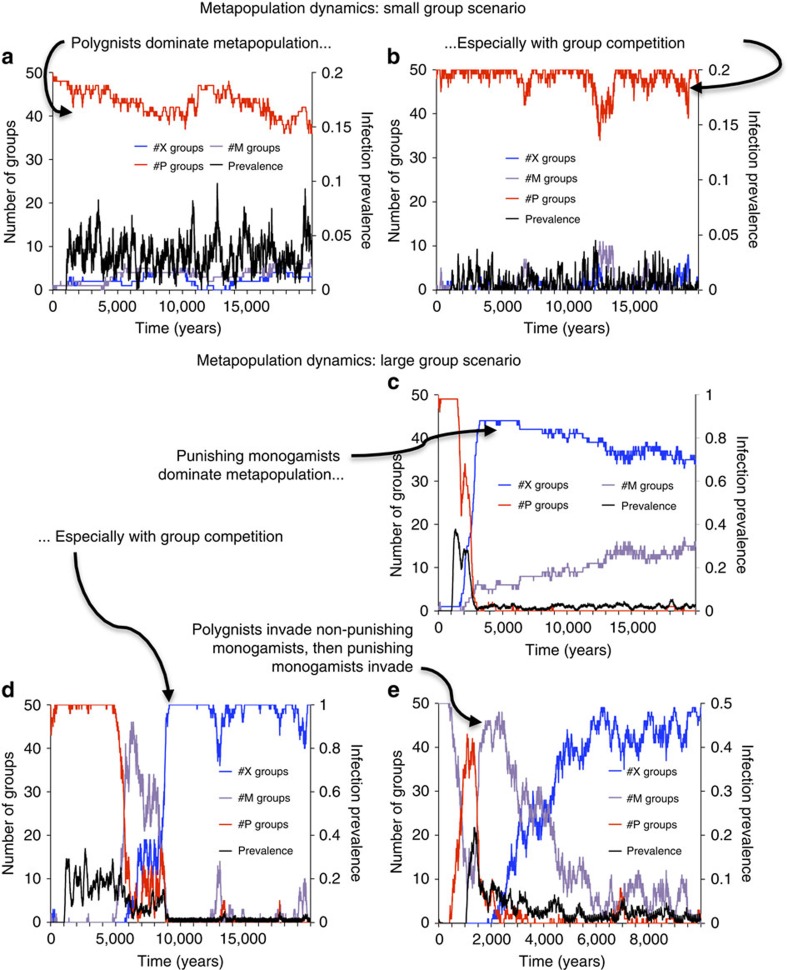
STIs become endemic when groups become large and cause the metapopulation to become dominated by punishing monogamist groups. Panels show the metapopulation dynamics of the number of punishing monogamist *X* (blue), non-punishing monogamist *M* (purple), and polygynist *P* (red) dominated groups, and metapopulation infection prevalence (proportion infected; black). Panels show the small group scenario without group competition (**a**) and with group competition (**b**), as well as the large group scenario without group competition (**c**) and with group competition (**d**). Also shown are metapopulation dynamics when the metapopulation is initially dominated entirely by monogamist groups, for the large group scenario with group competition (**e**). Metapopulation carrying capacity is 50 groups for all scenarios.

In the hunter–gatherer scenario where group sizes are small (group carrying capacity=30) and there is no group competition, we find that disease introductions cause short-lived outbreaks that impose a transient burden on polygynists. However, small group sizes ensure that stochasticity soon disrupts STI transmission, and the STI fades out. The remaining polygynists resurge in a disease-free environment (Fig. 1a). Hence, polygynists almost always dominate within groups because of their fertility advantage. Occasionally, monogamists or punishing monogamists can transiently dominate a group, but polygyny is eventually restored (Fig. 1b). At the metapopulation level, most groups are polygynist-dominated, although a few monogamist groups persist (Fig. 2a). Adding group competition simply reinforces this effect, consolidating the domination of polygynists, while also reducing infection prevalence by causing smaller average group sizes (Fig. 2b). While polygynists experience higher average STI prevalence than monogamists, it is negligible overall (Table 2).

In contrast, in the agriculturalist scenario where group sizes are larger (group carrying capacity=300), the STI becomes endemic in polygynist groups, causing group depopulation (Fig. 1c). Punishing monogamists invade the group by taking advantage of windows of opportunity when polygynists have been reduced to such small numbers that monogamists do not need to expend significant resources in punishing (Fig. 1d). The growing number of punishing monogamists further suppresses polygyny in a positive feedback loop, leading to eventual domination of the group by punishing monogamists (Fig. 1c,d). Sometimes, non-punishing monogamists are able to invade and dominate a group, and they tolerate a higher prevalence of polygynists in their group than punishers would (Fig. 1e). However, at the metapopulation level, the endemic STI enables punishing monogamists to dominate most groups, while monogamists persist at intermediate frequencies and purely polygynist groups are strongly suppressed (Fig. 2c). Group competition reinforces the dominance of punishing monogamists at the expense of non-punishing monogamists (Fig. 2d). As a result, infection prevalence decreases still further, compared with the scenario without group competition (Fig. 2d versus 2c, [Fig f2]).

A metapopulation consisting entirely of non-punishing monogamist groups can always be invaded by polygynists, who take advantage of low disease prevalence and lack of punishment (Fig. 2e). The result is a mixed metapopulation comprising mostly non-punishing monogamist groups, with a significant minority of polygynist groups. However, this state is short lived since punishing monogamist groups can subsequently invade (Fig. 2e). Hence, a purely non-punishing monogamous population cannot outcompete a mixed population of punishing and non-punishing monogamist groups.

Individual polygynists suffer higher STI prevalence than non-polygynists in their group, and polygynist groups suffer higher STI prevalence than non-polygynist groups (Table 2). This applies significant within-group and between-group selection against polygyny in the large group scenario. STI pressure ensures that polygynist groups remain smaller than punishing monogamist groups over the long term (Table 2). The size discrepancy between polygynist and monogamist groups favours monogamists under group competition.

### Probabilistic sensitivity analysis

These results are confirmed by a probabilistic sensitivity analysis, which evaluated the sensitivity of our results to simultaneous changes in multiple parameter values away from their baseline values. Triangular distributions were defined around the baseline parameter values (Table 1). We conducted 2,000 simulation runs, each lasting 30,000 years of simulated time. For each simulation run, parameter values were sampled from each of the triangular distributions described in Table 1. We found that punishing monogamist groups dominated the metapopulation in 59% of cases (that is, there were more punishing monogamist groups than either of the other two types by the end of the simulated time period), whereas non-punishing monogamist groups dominated in 13% of cases and polygynist groups dominated in 28% of cases.

### Univariate sensitivity analysis

These results are also confirmed by univariate sensitivity analysis on 14 model parameters (Supplementary Figs 2–15). The proportion of groups in the metapopulation; average group size; average individual fitness; and average prevalence (all stratified by strategy) were plotted as each of the 14 parameters was varied across a range one at a time, while the other parameters remained at their baseline values. Fifty realizations were conducted at each of the 10 tested parameter values across a given range.

The univariate sensitivity analysis also showed that the model outcomes are most sensitive to the frequency of group competition, the duration of infection and the proportion of total offspring provisioning provided by males.

Excessive group competition suppresses all groups and depopulates the entire metapopulation, while insufficient group competition removes the group-level benefits of socially imposed monogamy, although the within-group benefits are still sufficient to sustain monogamy and socially imposed monogamy as the primary strategies (Supplementary Fig. 8). A sufficiently long duration of infection imposes a heavy burden on the metapopulation, significantly suppressing or eliminating all groups, while a very short duration of infection reduces infection prevalence to such low levels that polygyny is no longer disadvantaged relative to monogamy (Supplementary Fig. 14). However, the resulting infection prevalence is unrealistically low compared with what is observed in real populations^47^. Finally, if males do not need to provision their offspring very much (such that females provide the great majority of resources), then polygyny confers sufficient fitness benefit to enable it to resist invasion by monogamists, although the parameter values at which this occurs correspond to unrealistically low levels of male provisioning (Supplementary Fig. 9).

### Quorum sensing and STI-induced mortality scenarios

We conducted additional univariate sensitivity analyses for scenarios where punishing monogamists do not apply punishment until their frequency in the group exceeds a certain threshold (quorum sensing; Supplementary Fig. 15) and where the STI causes mortality (Supplementary Fig. 3). Results are not sensitive to the quorum-sensing scenario, since increasing thresholds only further strengthens domination by punishing monogamists. Results are sensitive to sufficiently large STI mortality rates; however, the required mortality rate (10% probability of dying in the first year) is unrealistically high for bacterial STIs such as gonorrhea and chlamdyia, and in the case of syphilis, death does not occur until later stages^48^.

### Coital dilution scenario

In populations with concurrent partnerships, coital frequencies may be reduced in pairs where one of the individuals has other partners (‘coital dilution')^57^,^58^,^59^. Hence, we explored a scenario where polygynists have a reduced transmission rate because of coital dilution. We find that realistic parameter ranges for coital dilution do not change our predictions, and punishing monogamists are still able to invade because of greater STI-imposed burden on polygynists (Supplementary Fig. 16 and Supplementary Methods).

### Chronic infection scenario

We also explored a scenario for a chronic infection with a low transmission rate, such as HIV. We choose parameters based on HIV infection for the infertility rate *θ*=0.5 (ref. 21), transmission probability *τ*=0.04 per month (ref. 60), and disease-induced death probability *μ*=0.1 per year (ref. 61; Supplementary Methods). At baseline parameter values, the results are similar to the bacterial STI scenario (Supplementary Table 2). Under the probabilistic sensitivity analysis, punishing monogamist groups dominated the metapopulation 71% of the time, monogamists dominated 25% of the time and polygynist groups dominated only 4% of the time. However, the results for the chronic infection scenario are much more sensitive to coital dilution at empirically realistic levels of coital dilution (Supplementary Fig. 17 and Supplementary Methods). This confirms the intuition that network concurrency should be more important for infectious diseases with high transmission rate and short duration of infection than for diseases with low transmission rate and long duration of infection^56^,^62^.

## Discussion

Here we developed a simulation model based on known demographic profiles of hunter–gatherers and agriculturalists and bacterial STI epidemiology, and we used the model to show how growing STI disease burden in larger residential group sizes can foster the emergence of socially imposed monogamy in human mating through processes of cultural evolution.

Our hypothesis is compatible with other hypotheses on the emergence of socially imposed monogamy^12^,^13^,^14^,^15^,^16^,^17^,^18^. For example, other effects of monogamy (such as increased paternal investment and reduced crime rates and intrahousehold conflict) have been hypothesized to boost the fitness of monogamous groups under group competition^15^. Alternatively, the within-group inclusive fitness benefits of monogamy may have provided an initial impetus for monogamy to get started^18^, after which the group-level and individual-level mechanisms we explore begin to augment the advantages of socially imposed monogamy, as residential group sizes increased. As noted in the Introduction, multiple mechanisms may have been involved in the emergence of socially imposed monogamy, since social norms are a complex phenomenon.

We designed our model with conservative assumptions so that it was more difficult for punishing monogamists to invade. For instance, punishing monogamy had to be able to invade only with the benefit of differential STI prevalence, although in reality, multiple effects could have acted together to promote monogamy^12^,^13^,^14^,^15^,^16^,^17^,^18^. In addition, we did not allow punishing monogamists to engage in quorum sensing, although in reality, punishing monogamists would be capable of quorum sensing. Third, we assumed a conservative value for the provisioning factor *f* such that males provided significantly less support to offspring than females, although in reality this factor is higher^37^,^39^. Finally, we assumed a simple strategy set where individuals could adopt only (punishing or non-punishing) monogamist or polygynist strategies. However, a larger strategy set where individuals adopt a strategy *n*, where *n* is their maximum number of partners, might make it easier for socially imposed monogamy to invade by allowing for bridge strategies between monogamy and unconstrained polygyny.

We made other simplifying assumptions that could have an impact on model predictions. For instance, we assumed females always adopt a single strategy of sorting mates by an ideal free distribution, whereas future work could introduce a strategy set for females. In addition, we note that mating and marriage are distinct (although related) phenomena in humans, and our model concerns norms for restraint in mating, not marriage *per se*. Previous models of STI transmission and control have distinguished between short-term casual partnerships and long-term steady partnerships^41^. This previous work could form a basis for future models that distinguish explicitly between marriage and mating.

Moreover, there is little data on STI prevalence in hunter–gatherer and agriculturalist populations before the advent of germ theory and modern testing, treatment and prevention. Perhaps, the earliest reliable reference to gonorrhea discusses ‘the perilous infirmity of burning' in the 1161 Acts of the (English) Parliament^52^, although biblical references to what appears to be gonorrhea also occur^63^. Syphilis existed for certain by the Fifteenth century, although there is debate about whether its origin was Colombian or pre-Colombian^63^,^64^. Future work could attempt to overcome these challenges and undertake an empirical test of our hypothesis. Because of the lack of data on STIs in prehistoric hunter–gatherer populations, data from contemporary populations could be used. Whether polygyny is a harmful or beneficial cultural practice at various levels of health and well-being is a topic of continuing interest in the literature^65^. Recent research has evaluated the relationship between polygyny and HIV prevalence in contemporary African populations. Different analyses have come to different conclusions about whether polygyny increases HIV prevalence^28^,^55^,^56^,^57^. One such analysis found that HIV prevalence is strongly and positively associated with extramarital sexual partners, but weakly and negatively associated with being in a polygynous marriage^56^. This finding underlines the need to distinguish between marriage and mating in future work. However, the differing natural history of HIV versus bacterial STIs has significant implications for the impact of concurrency on STI prevalence^56^,^62^; therefore, it is difficult to extrapolate convincingly from HIV-focused studies to our research question. An empirical test of our hypothesis would require carefully controlled comparisons that account for confounding variables such as availability of prevention methods (for example, latex condoms), testing and treatment that were not available in hunter–gatherer populations, as well as the effects of cultural phylogeny. Interventions such as latex condoms could influence the dynamics of our model. However, they are not directly relevant to our hypothesis, which concerns a period of human prehistory and early history for which there is little evidence of widespread condom use.

Related to the issue of marital institutions and the need to distinguish between marriage and mating in future work is the role of religion. Constraints on sexual behaviour are often closely associated with religious beliefs and institutions. The injunctive social norms we explore here are naturally operationalized and institutionalized as religiously decreed constraints on polygyny, as occur in Christianity for instance. Future work could explicitly include the role of religion in a way similar to how marital institutions could be included.

Finally, the sexual network aspect of the model could be further explored in future work, and related to existing work on network theory and STI transmission through sexual networks. Analytical methods such as percolation theory^66^ or pair approximation^25^,^67^ may yield closed-form analytical expressions describing conditions for the emergence of socially imposed monogamy on sexual partnership networks, although stochasticity complicates the analysis. Previous modelling approaches that investigate STI transmission and control have used everything from deterministic ordinary differential equation models without^29^ durable partnerships, to deterministic ordinary differential equation models with partnerships of a specified duration that can^25^ or cannot^68^ account for overlapping partnerships, to agent-based sexual network models with completely specific sexual partnership networks^26^,^28^,^41^,^55^,^69^. Approaches using fully specified agent-based network models have explored interventions for the control of chlamydia^41^,^42^,^69^,^70^ and gonorrhoea^41^; the role of concurrency in the spread of HIV^26^,^28^,^55^; the impact of network heterogeneities on STI transmission^67^; and the use of network models (and also compartmental models) to estimate important epidemiological parameters such as transmission rates^51^,^67^. In our model, females only mate with one male and therefore the total number of partnerships is the same, on average, between polygynist groups and monogamist groups. Therefore, our model is most similar to previous models that explore the effect of concurrency on HIV transmission by changing concurrency without altering the overall number of partnerships^25^,^26^.

In summary, our model illustrates a potential pathway that might help explain the emergence—and timing of emergence—of socially imposed monogamy resulting from interactions between residential group size, STI dynamics and their impact on fertility, and the evolutionary dynamics of social norms. Our model couples natural and social dynamics and therefore complements approaches that explore costly punishment evolution using models with fixed payoff matrices^8^. In our model, multilevel selection is endogenously regulated by disease dynamics rather than depending on fixed payoff matrices. Approaches that couple natural and social dynamics can provide insights regarding not only the emergence of socially imposed monogamy, but also other social norms relating to human physical contact.

## Methods

### Software

We developed an event-driven, discrete-time agent-based simulation model, coded in the C programming language (Supplementary Data File).

### Strategies

Males can play the strategy P (polygynous), M (monogamous) or X (monogamous punisher). M and X strategists will not accept more than one mate at a time (serial monogamy is allowed), whereas P strategists do not limit how many mates they can have at any point in time. Females only have one strategy of seeking any available male and do not prefer P, M or X (in reality, females will strategically sort according to an ideal free distribution; we address this in sensitivity analysis). At any given time, individuals are either in a pair (with at least one mate) or single (no mates).

### Mate selection dynamics

Single females look for mates with probability *ρ* per timestep, picking an available male (either a single M or X strategist, or any P strategist) at random, who then becomes their mate. With probability *ɛ,* a single female looks for mates in other groups and move to that group if they find an available mate (representing exogamy), and otherwise she looks for mates within their own group. A partnership breaks up with probability *ω* per timestep.

### Vital dynamics

Individuals die because of non-STI-related causes at rate *δ* per timestep. Those whose mate has died become single and seek a new mate according to the usual mate selection dynamics. Each pair has an offspring with probability *β* per timestep, where *β* is sampled from a lognormal probability distribution. The birth rate in a pair where the male has other mates is reduced by a factor 1/(1−φ+φ*N*), where *N* is the number of mates of the male and φ is called the provisioning factor, representing what contribution the male makes to provision the family. The offspring is female with a 50% chance, and male otherwise. Male offspring adopt a strategy through a social learning process. They (1) randomly select a male in the group, and (2) if the sampled male is receiving a higher payoff than the average male payoff in the group, they adopt the sampled male's strategy with a probability *L*/(1+*L*) where *L* is the incremental difference in payoff between the sampled male and the average male in the group; if a strategy is not found, they go back to step 1 and repeat until a strategy has been chosen. Hence, a newly recruited individual is more likely to adopt a strategy if it is more prevalent, and if it is more successful. There is a sampling error; therefore, some fraction *σ* of new recruits picks a strategy randomly. The payoff of an individual is their current lifetime number of offspring. Once individuals have chosen a strategy, they do not change it.

### STI emergence and spread

Every Δ timesteps, each individual has a probability *π* of becoming the index case of a new outbreak of a STI (hence, as the population grows, the rate of emergence of new infections increases). The STI spreads from an infected individual to a susceptible partner, with probability *τ* per timestep. Moreover, a proportion *θ* of infected individuals do not have further offspring because of infertility or stigma. Individuals clear the infection and become susceptible again, with a probability *λ* per unit time. We neglect vertical transmission.

### Punishment

In every timestep, each X strategist in a group pays a cost *c* to incur a penalty *c* on each P strategist in the group. The cost and penalty of punishment are measured in reduced birth rate, such that the birth probability *β* of a pair involving an X strategist is multiplied by a factor (1−*cP*), and the birth probability of a pair involving a P strategist is multiplied by a factor (1−*cX*). We constrain birth probabilities to be non-negative.

### Group dynamics

Each group initially consists of 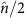 males and 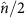 females. At *t=*0, all individuals are initially single, and seek out mates according to the usual mate selection process. Overall, 96% of males are initially assigned to be P strategists, 2% are X strategists and 2% are M strategists. There is a maximum number of possible groups *N*_max_, and the number of groups starts out at *N*_max_ but may drop below that because of mortality. Group size is variable, being a function of births and deaths; however, each group has a carrying capacity *K*, and no further births are possible in a group that has reached size *K*. The initial population size is 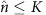 for all groups. When a group's size reaches size 0.9 *K*, the group divides into two groups as long as there is an available space for a daughter group to be formed (that is, as long as *N*<*N*_max_). Each male is assigned to the daughter group with a 50% probability, and otherwise stays in the mother group. Existing pairs are conserved so that females move with their mates to the new group, if applicable. Any remaining single females remain in the mother group.

A group selection mechanism is also applied. In each timestep, with probability *κ*, two randomly selected groups compete. The probability that group A is victorious over group B is 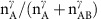, where *n*_A_ and *n*_B_ are their respective group sizes. The factor *γ* controls the extent to which factors besides group size determine the victorious group. The vanquished group and all of its members are removed completely and half of the males of the victorious group take over the spot of the vanquished group, along with their mates.

## Additional information

**How to cite this article:** Bauch, C. T. & McElreath, R. Disease dynamics and costly punishment can foster socially imposed monogamy. *Nat. Commun.* 7:11219 doi: 10.1038/ncomms11219 (2016).

## Supplementary Material

Supplementary Information Supplementary Figures 1-17, Supplementary Tables 1-2, Supplementary Methods and Supplementary References

Supplementary Software Agent-based Simulation Code

## Figures and Tables

**Table 1:** Model parameters, baseline values, intervals for probabilistic sensitivity analysis and literature sources.

**Parameter**	**Description**	**Baseline value and PSA** **intervals**	**Source**
*ρ*	Probability of seeking partner per unit time	0.1 per month	(33)
*ɛ*	Probability of practicing exogamy	0.8 (0.6, 1.0)	(34)
*ω*	Probability of pair breakup per unit time	0.05 per year (0.04, 0.06)	(35)
*δ*	Probability of non-STI related death per unit time	0.01429 per year	(36)
*β*	Mean birth probability per unit time	0.079 per year	(37)
*s*	Scale parameter controlling standard deviation of lognormal birth probability	0.29 (0.23, 0.35)	(37)
*χ*	Mate selection exponent	1.5 (1.2, 1.8)	(38) (calibrated)
φ	Provisioning factor	0.3 (0.2, 0.4)	(37, 39)
*σ*	Fraction of recruits picking strategy randomly	0.01	Assumption
Δ	Time between disease re-introductions	70 years (40, 100)	Assumption
*π*	Probability of being an index case	0.01	Assumption
*τ*	Probability of STI transmission in a pair per unit time	0.85 per month (0.8, 0.9)	(40–43, 51)
*λ*	Probability of clearing infection	1.0 per year (0.8, 1.2)	(44–47, 49)
*θ*	Probability that infection sterilizes or causes pregnancy loss	0.05 (0.04, 0.06)	(24, 48, 50)
*c*	Cost of punishing cost of being punished	0.01 (0.005, 0.015)	Assumption
*N*_max_	Maximum number of groups	50	Assumption
*K*	Group carrying capacity	Small group: 30 Large group: 300	(37)
*κ*	Probability of group competition per unit time	No comp: 0 per month Comp: 0.05 per month (0.03, 0.07)	Assumption
*γ*	Factor determining importance of group size in competition events	10 (5, 15)	Assumption

PSA, Probabilistic Sensitivity Analysis; STI, sexually transmitted infection.

**Table 2:** STI prevalence and group size by strategy*.

	**Small group scenario**				**Large group scenario**			
	**Within X groups**	**Within M groups**	**Within P groups**	**Across all groups**	**Within X groups**	**Within M groups**	**Within P groups**	**Across all groups**
STI prevalence								
All individuals	0.003 (±0.003)	0.002 (±0.003)	0.004 (±0.0003)	0.004 (±0.0003)	0.014 (±0.035)	0.038 (±0.024)	0.181 (±0.042)	0.029 (±0.018)
X individuals	0.002 (±0.002)	0.001 (±0.004)	0.001 (±0.001)	0.001 (±0.001)	0.01 (±0.014)	0.017 (±0.01)	0.075 (±0.024)	0.013 (±0.007)
M individuals	0.002 (±0.009)	0.001 (±0.002)	0.001 (±0.001)	0.001 (±0.001)	0.008 (±0.001)	0.024 (±0.012)	0.083 (±0.02)	0.019 (±0.007)
P individuals	0.009 (±0.016)	0.007 (±0.012)	0.004 (±0.0003)	0.004 (±0.0003)	0.082 (±0.088)	0.171 (±0.036)	0.228 (±0.063)	0.129 (±0.019)
Group size	21.3 (±0.8)	21.5 (±0.7)	22.4 (±0.1)	22.3 (±0.1)	238.2 (±27.1)	231.1 (±21.9)	199.4 (±27.5)	222.4 (±27.3)

STI, sexually transmitted infection.

^*^Long-term average group size and STI prevalence by strategy type, in monogamist (X), non-punishing monogamist (M) and polygynists (P) groups and across the whole population, for the small group and large group scenarios. Values are the average across 100 simulation runs over 30,000 years each, and parenthetical values denote one s.d.
