# Supplementary material for: Disease dynamics and costly punishment can foster socially imposed monogamy
Source: Nat Commun. 2016 Apr 12;7:11219. doi: 10.1038/ncomms11219 (PMC4832056; doi:10.1038/ncomms11219)
Supplement: Supplementary Information — Supplementary Figures 1-17, Supplementary Tables 1-2, Supplementary Methods and Supplementary References [file ncomms11219-s1.pdf]

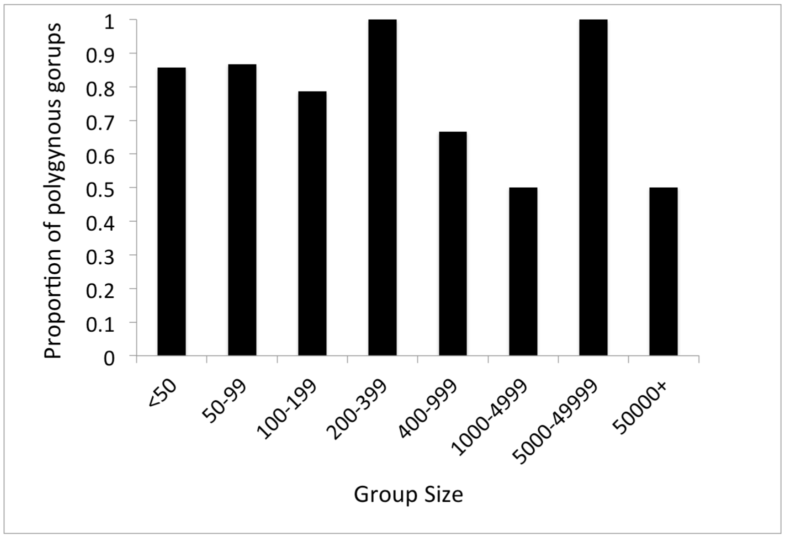

Supplementary Figure 1: Proportion of polygynous groups versus group size categories. Data from Supplementary Table 1.

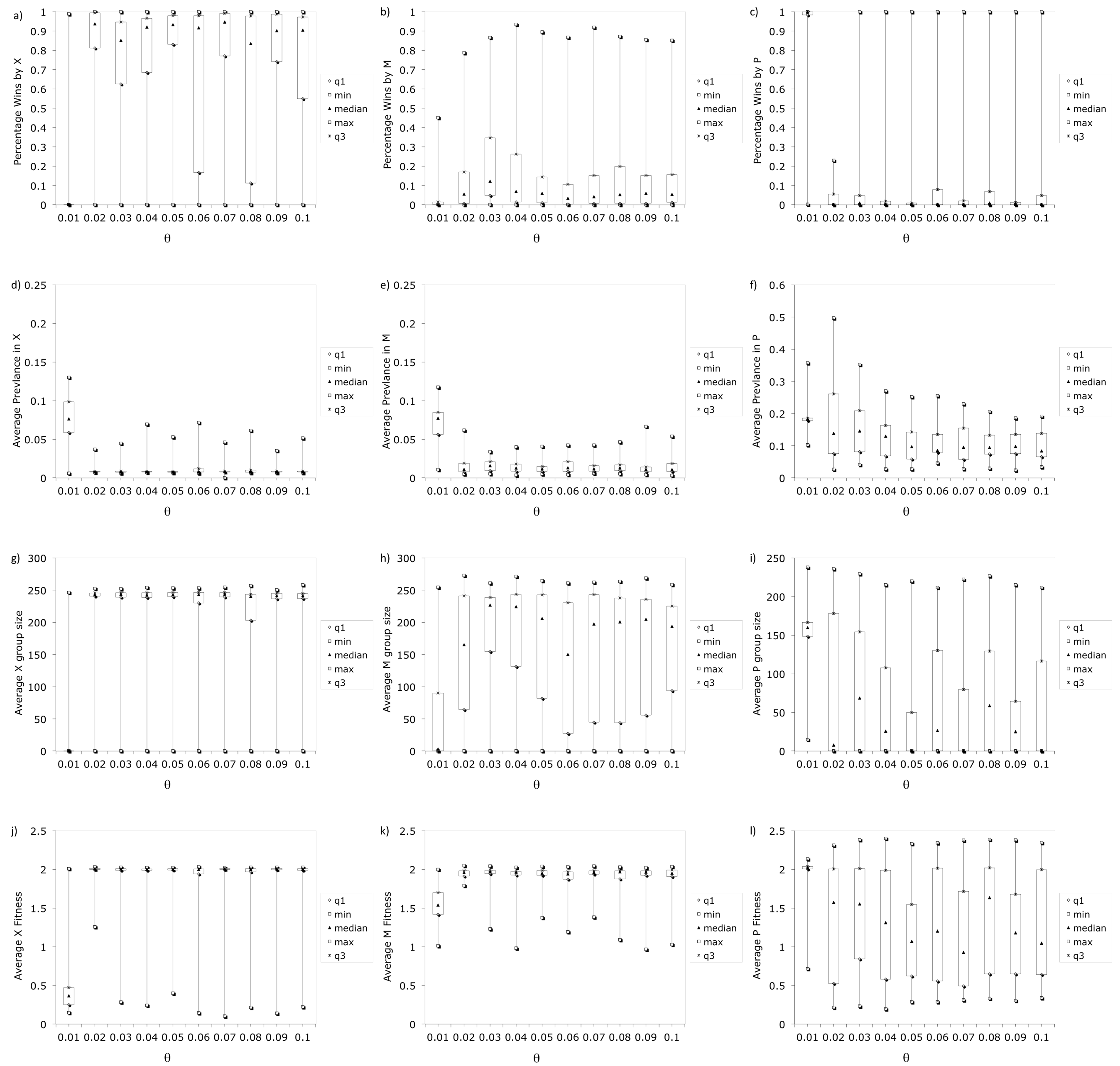

<u>Supplementary Figure 2</u>: **Results are insensitive to rates of infertility caused by STIs, except when rates are very low (thus making the infection harmless).** Univariate sensitivity analysis with respect to θ, the percentage of infected persons made infertile, for: percentage of groups at end of simulation dominated by X, M and P individuals **(a-c)**; average infection prevalence in X, M and P individuals **(d-f)**; average size of groups dominated by X, M and P individuals **(g-i)**; and average fitness of X, M and P individuals **(j-k)**. 'X' denotes punishing monogamists, 'M' denotes non-punishing monogamists, 'P' denotes polygynists. Boxplots show median (▲), first quartile (◇), third quartile (*), minimal value observed (□) and maximal value observed (□).

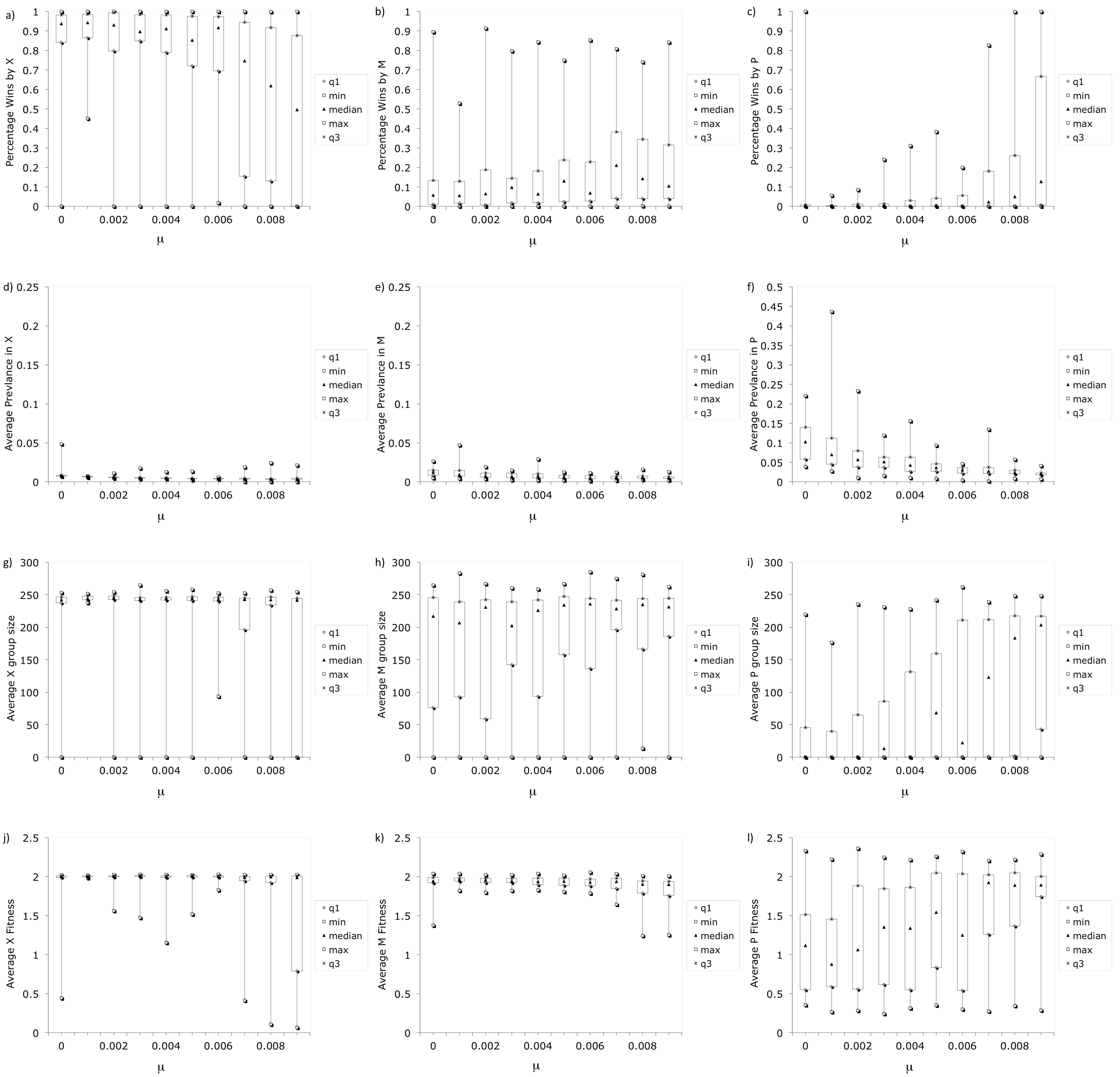

<u>Supplementary Figure 3</u>: **Increasing STI mortality rates lower STI prevalence in polygynists, thus making them more competitive.** Univariate sensitivity analysis with respect to μ, the percentage of infected persons dying from infection, for: percentage of groups at end of simulation dominated by X, M and P individuals **(a-c)**; average infection prevalence in X, M and P individuals **(d-f)**; average size of groups dominated by X, M and P individuals **(g-i)**; and average fitness of X, M and P individuals **(j-k)**. 'X' denotes punishing monogamists, 'M' denotes non-punishing monogamists, 'P' denotes polygynists. Boxplots show median (▲), first quartile (◇), third quartile (*), minimal value observed (□) and maximal value observed (□).

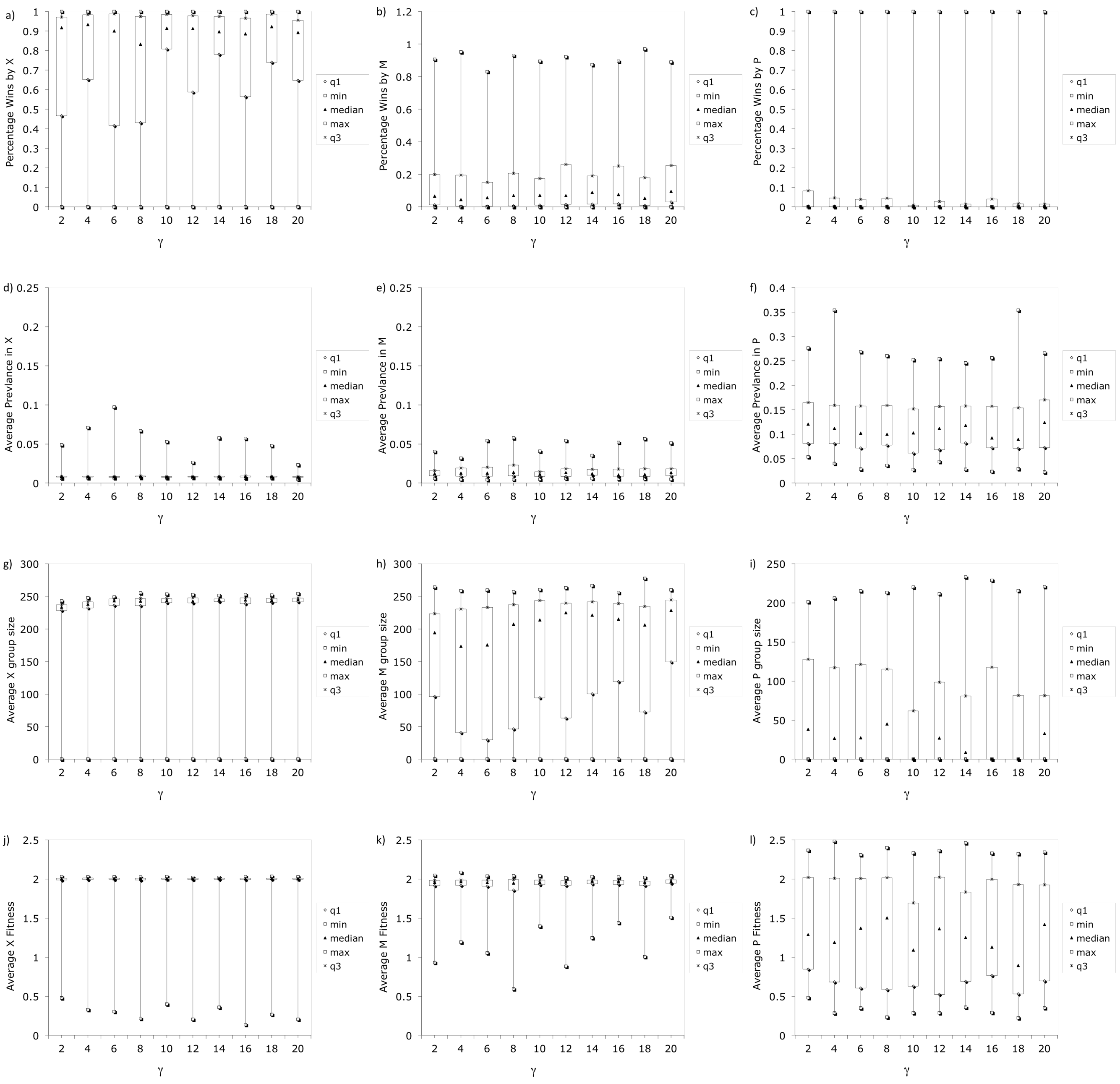

<u>Supplementary Figure 4</u>: **Results are insensitive to how strongly group size determines group competition outcomes.** Univariate sensitivity analysis with respect to ***γ***, the factor determining whether group size or stochasticity determines the winner of group competition contests, for: percentage of groups at end of simulation dominated by X, M and P individuals **(a-c)**; average infection prevalence in X, M and P individuals **(d-f)**; average size of groups dominated by X, M and P individuals **(g-i)**; and average fitness of X, M and P individuals **(j-k)**. 'X' denotes punishing monogamists, 'M' denotes non-punishing monogamists, 'P' denotes polygynists. Boxplots show median (▲), first quartile (◇), third quartile (*), minimal value observed (□) and maximal value observed (□).

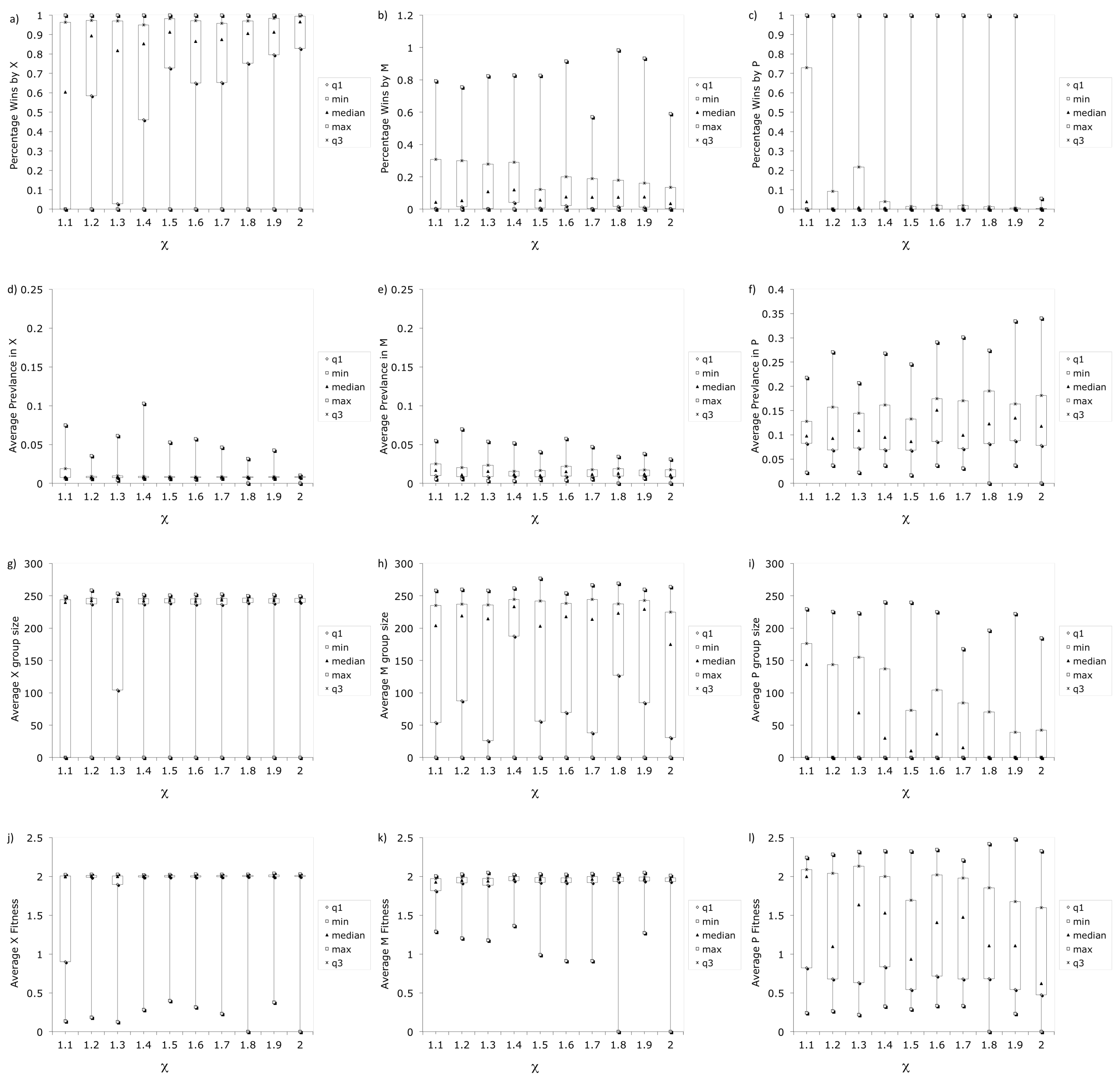

<u>Supplementary Figure 5</u>: Results are insensitive to factor controlling the desirability of polygynists with multiple mates (note that values sufficiently different from the baseline value χ=1.5 yield unrealistic distributions of number of partners among polygynists). Univariate sensitivity analysis with respect to χ, the mate selection exponent, for: percentage of groups at end of simulation dominated by X, M and P individuals **(a-c)**; average infection prevalence in X, M and P individuals **(d-f)**; average size of groups dominated by X, M and P individuals **(g-i)**; and average fitness of X, M and P individuals **(j-k)**. 'X' denotes punishing monogamists, 'M' denotes non-punishing monogamists, 'P' denotes polygynists. Boxplots show median (▲), first quartile (◇), third quartile (✱), minimal value observed (□) and maximal value observed (□).

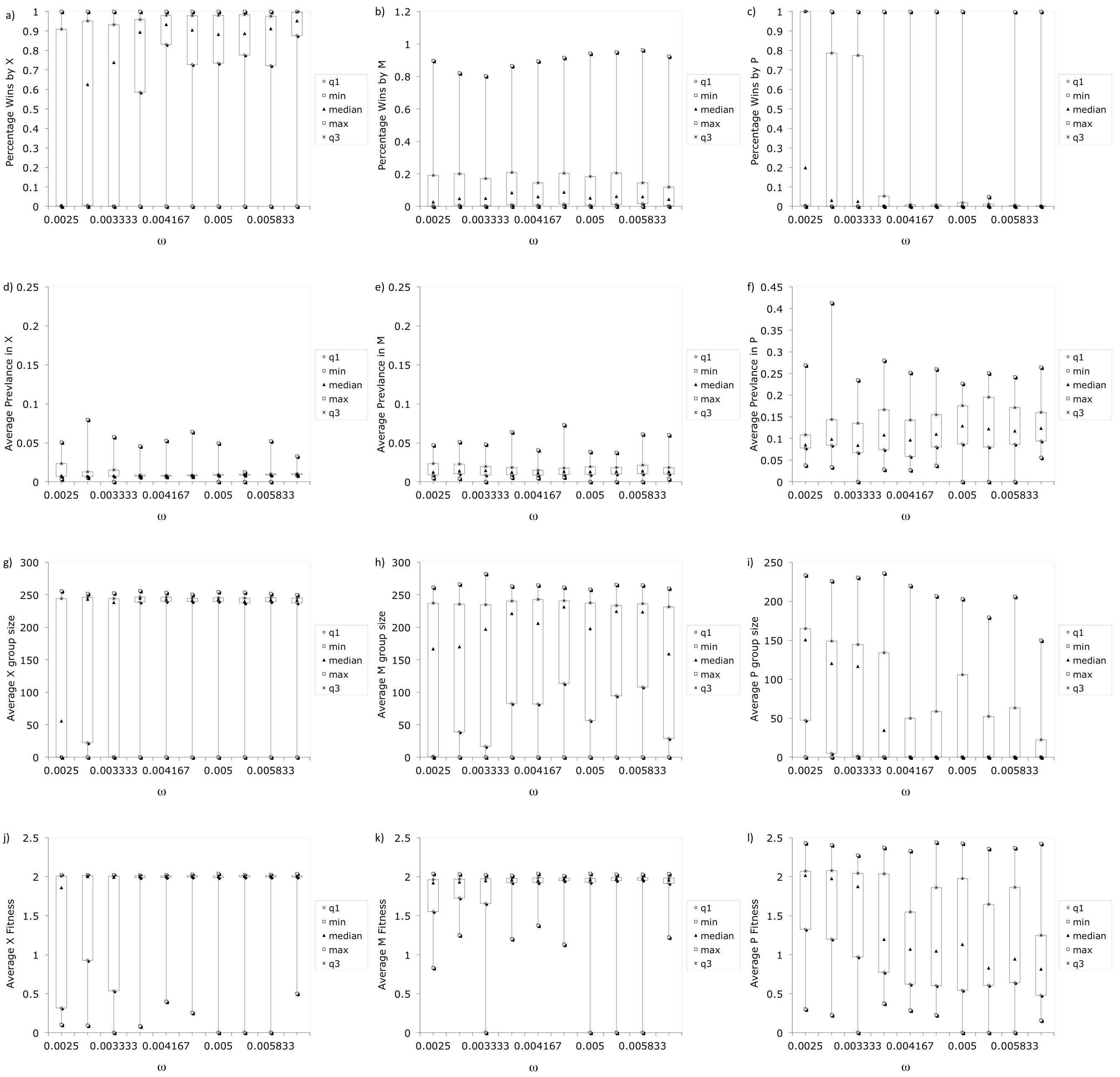

<u>Supplementary Figure 6</u>: Sufficiently long partnership duration (~30 years) prevents dominance of any given strategy. Univariate sensitivity analysis with respect to ω, the probability of partnership breakup per month, for: percentage of groups at end of simulation dominated by X, M and P individuals **(a-c)**; average infection prevalence in X, M and P individuals **(d-f)**; average size of groups dominated by X, M and P individuals **(g-i)**; and average fitness of X, M and P individuals **(j-k).** 'X' denotes punishing monogamists, 'M' denotes non-punishing monogamists, 'P' denotes polygynists. Boxplots show median (▲), first quartile (◇), third quartile (✱), minimal value observed (□) and maximal value observed (□).

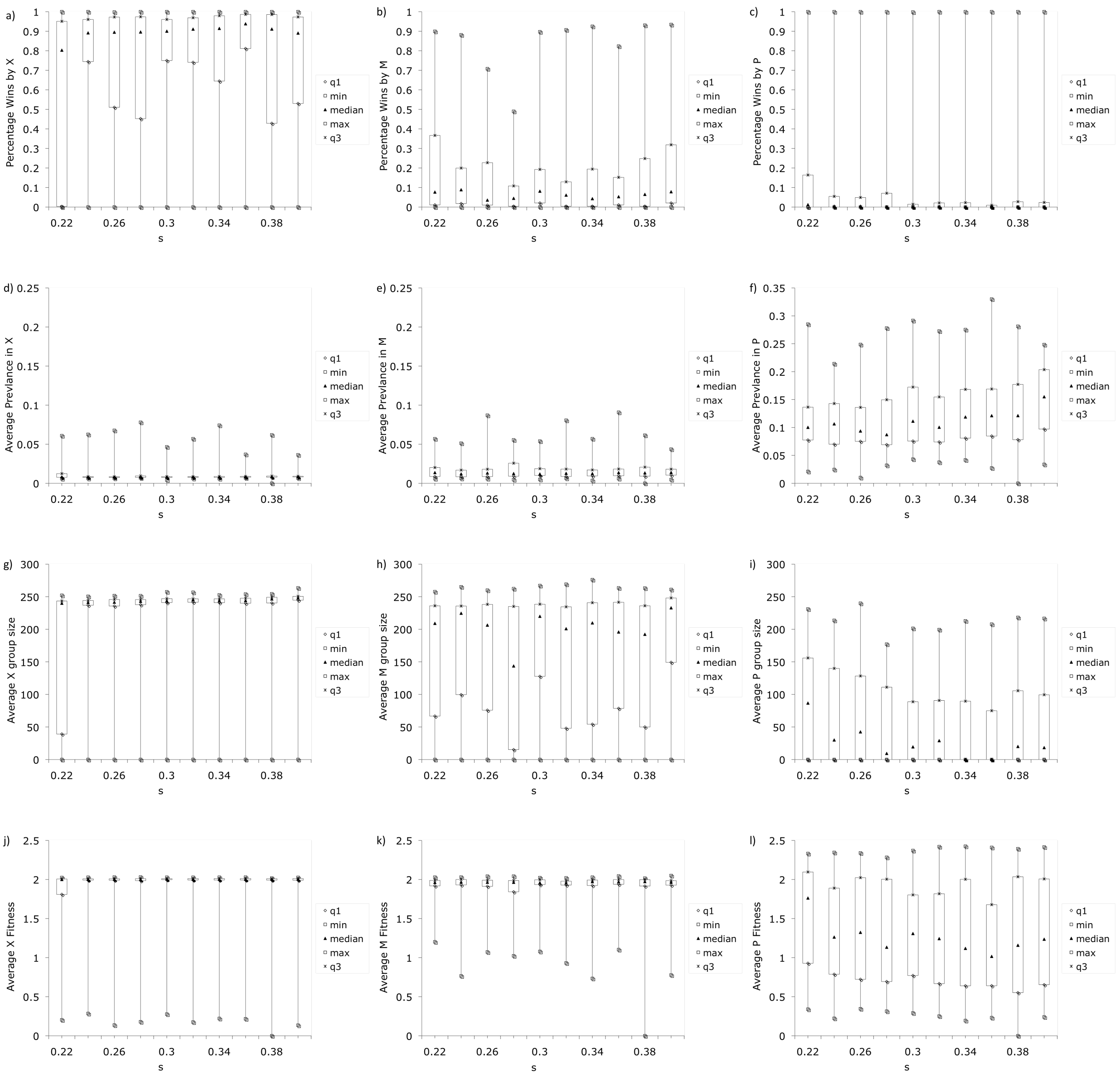

<u>**Supplementary Figure 7**</u>: **Results are not very sensitive to changes in birth rate.** Univariate sensitivity analysis with respect to s, the shape parameter for standard deviation of birth probabilities, for: percentage of groups at end of simulation dominated by X, M and P individuals **(a-c)**; average infection prevalence in X, M and P individuals **(d-f)**; average size of groups dominated by X, M and P individuals **(g-i)**; and average fitness of X, M and P individuals **(j-k)**. 'X' denotes punishing monogamists, 'M' denotes non-punishing monogamists, 'P' denotes polygynists. Boxplots show median (▲), first quartile (◇), third quartile (*), minimal value observed (◻) and maximal value observed (◻).

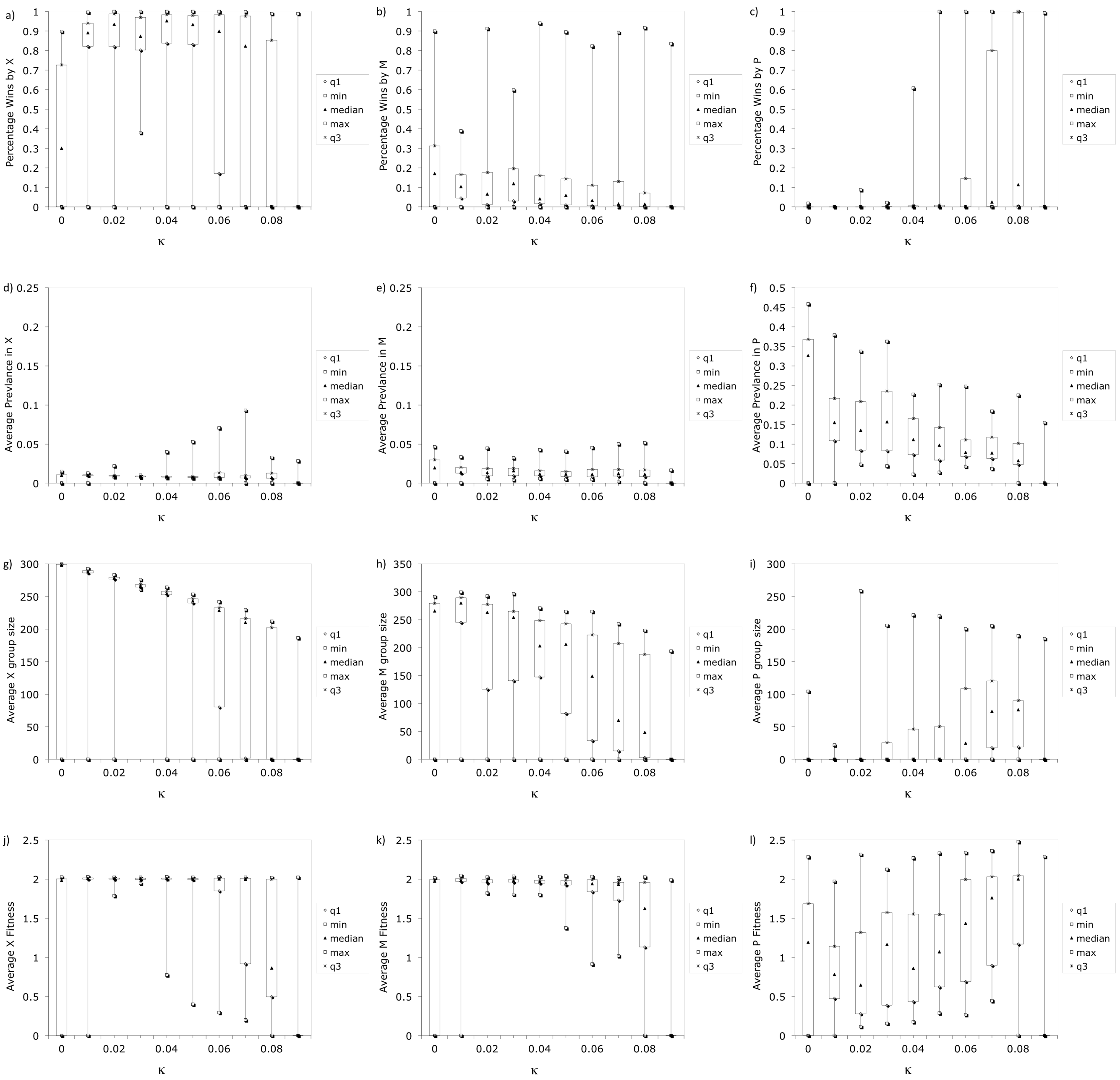

<u>Supplementary Figure 8</u>: **Too little group competition favours non-punishing monogamy, but too much destroys all groups.** Univariate sensitivity analysis with respect to κ, the probability of group competition per month, for: percentage of groups at end of simulation dominated by X, M and P individuals **(a-c)**; average infection prevalence in X, M and P individuals **(d-f)**; average size of groups dominated by X, M and P individuals **(g-i)**; and average fitness of X, M and P individuals **(j-k).** 'X' denotes punishing monogamists, 'M' denotes non-punishing monogamists, 'P' denotes polygynists. Boxplots show median (▲), first quartile (◇), third quartile (*), minimal value observed (□) and maximal value observed (□).

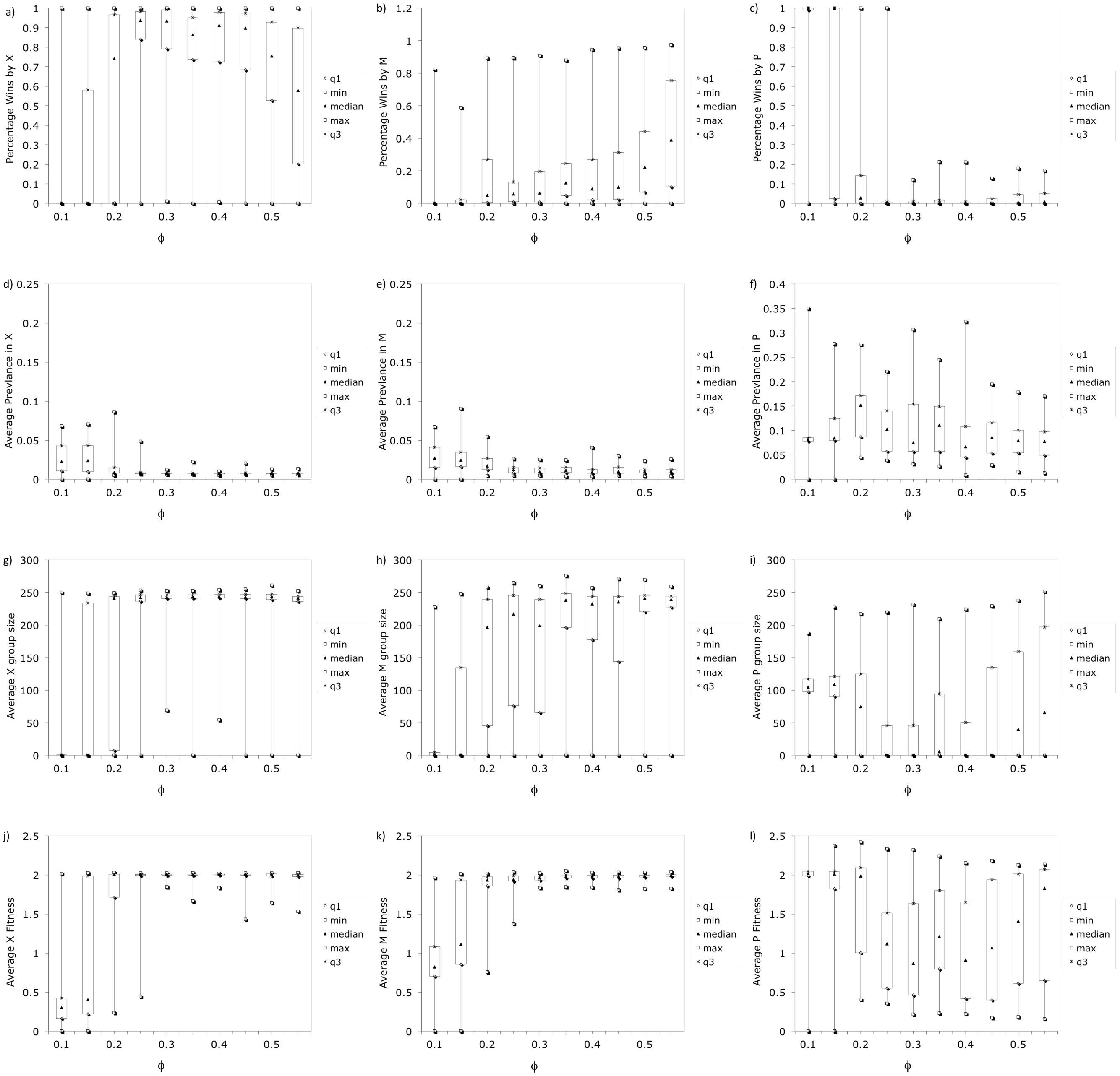

<u>Supplementary Figure 9</u>: Punishing monogamists cannot invade when polygynists do not need to provision their offspring. Univariate sensitivity analysis with respect to ϕ, the fertility penalty, for: percentage of groups at end of simulation dominated by X, M and P individuals **(a-c)**; average infection prevalence in X, M and P individuals **(d-f)**; average size of groups dominated by X, M and P individuals **(g-i)**; and average fitness of X, M and P individuals **(j-k)**. 'X' denotes punishing monogamists, 'M' denotes non-punishing monogamists, 'P' denotes polygynists. Boxplots show median (▲), first quartile (◇), third quartile (*), minimal value observed (□) and maximal value observed (□).

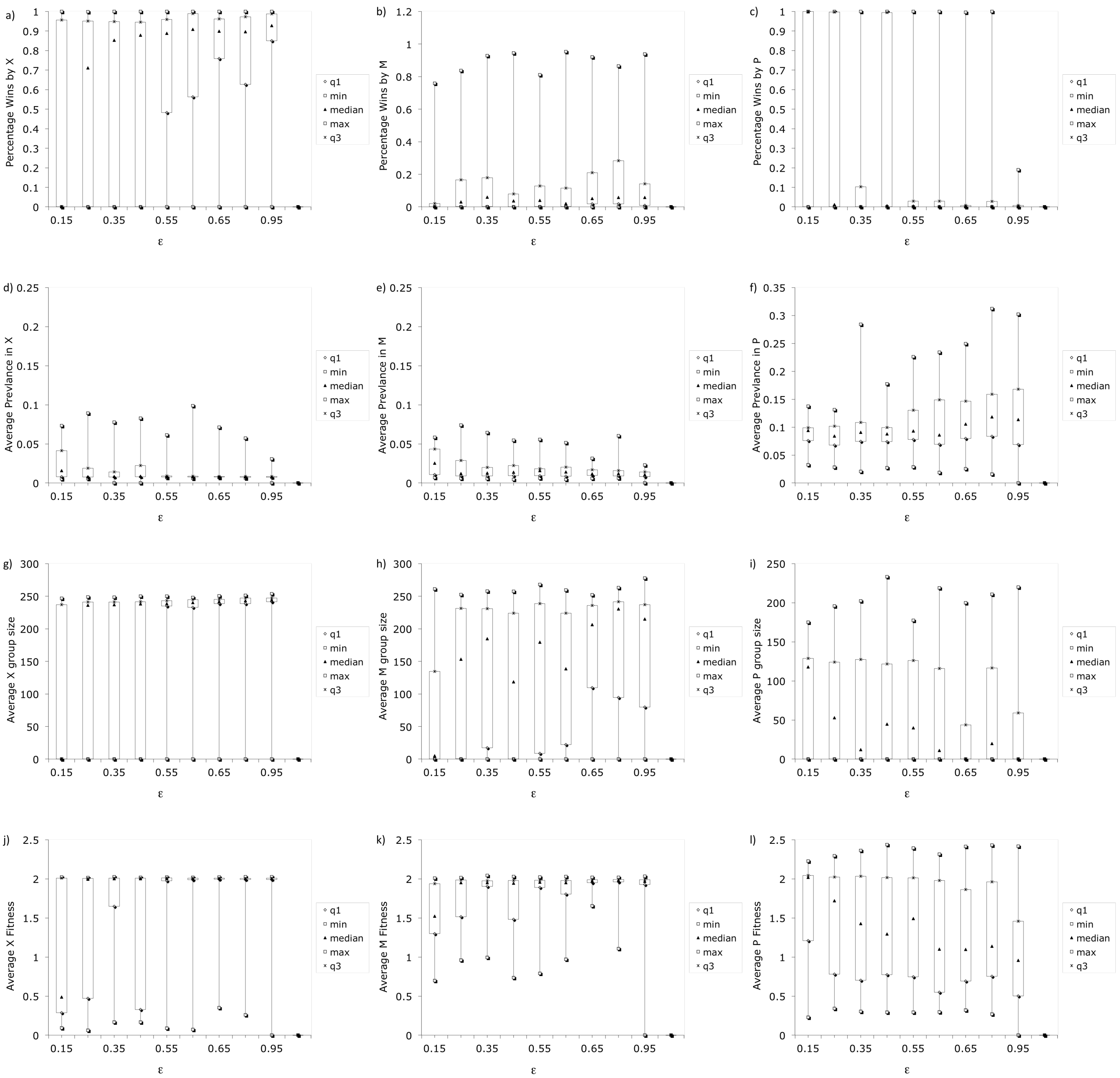

Supplementary Figure 10: **Very low rates of exogamy prevent monogamists from invading** (realistic rates exceed 50%). Univariate sensitivity analysis with respect to ε, the probability a feamel chooses exogamy, for: percentage of groups at end of simulation dominated by X, M and P individuals **(a-c)**; average infection prevalence in X, M and P individuals **(d-f)**; average size of groups dominated by X, M and P individuals **(g-i)**; and average fitness of X, M and P individuals **(j-k)**. 'X' denotes punishing monogamists, 'M' denotes non-punishing monogamists, 'P' denotes polygynists. Boxplots show median (▲), first quartile (◇), third quartile (*), minimal value observed (□) and maximal value observed (□).

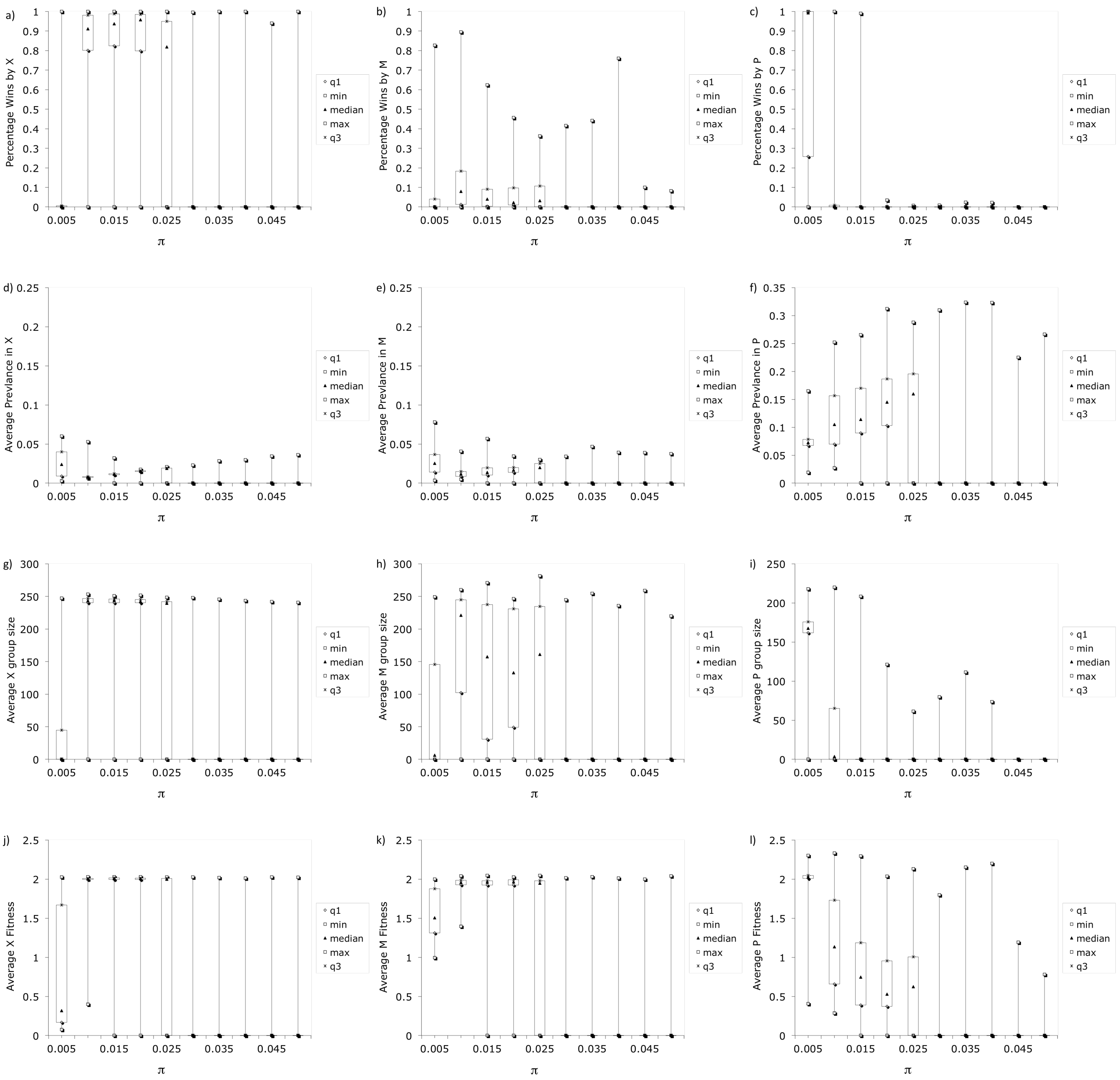

<u>**Supplementary Figure 11**</u>: **Sufficiently high case importation rates reduce all groups.** Univariate sensitivity analysis with respect to π, the probability of being an index case during case importation periods, for: percentage of groups at end of simulation dominated by X, M and P individuals **(a-c)**; average infection prevalence in X, M and P individuals **(d-f)**; average size of groups dominated by X, M and P individuals **(g-i)**; and average fitness of X, M and P individuals **(j-k)**. 'X' denotes punishing monogamists, 'M' denotes non-punishing monogamists, 'P' denotes polygynists. Boxplots show median (▲), first quartile (◇), third quartile (✱), minimal value observed (□) and maximal value observed (□).

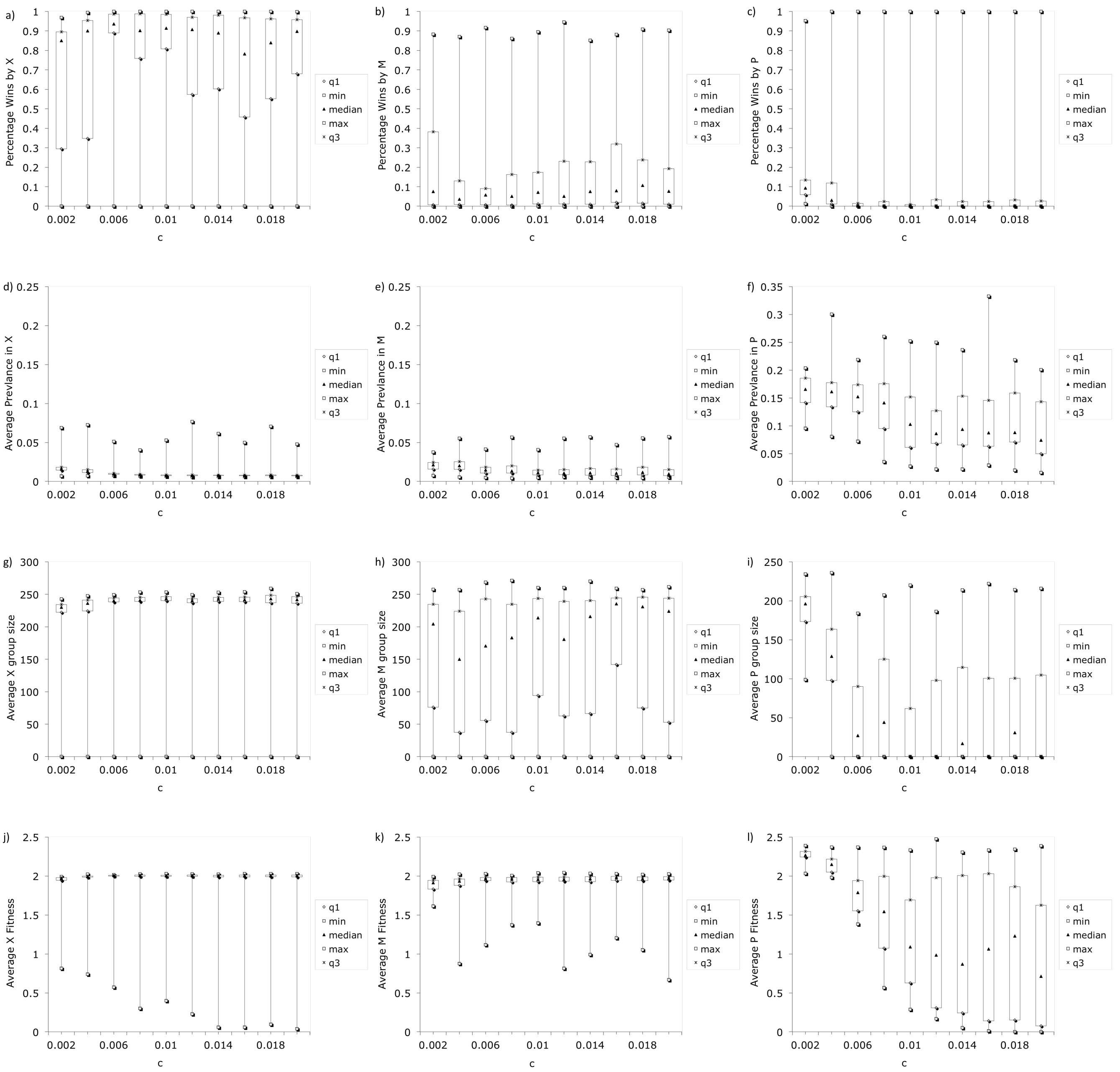

<u>Supplementary Figure 12</u>: Results are insensitive to the cost of punishing/being punished. Univariate sensitivity analysis with respect to c, the cost of punishing/being punished, for: percentage of groups at end of simulation dominated by X, M and P individuals **(a-c)**; average infection prevalence in X, M and P individuals **(d-f)**; average size of groups dominated by X, M and P individuals **(g-i)**; and average fitness of X, M and P individuals **(j-k)**. 'X' denotes punishing monogamists, 'M' denotes non-punishing monogamists, 'P' denotes polygynists. Boxplots show median (▲), first quartile (◇), third quartile (*), minimal value observed (□) and maximal value observed (□).

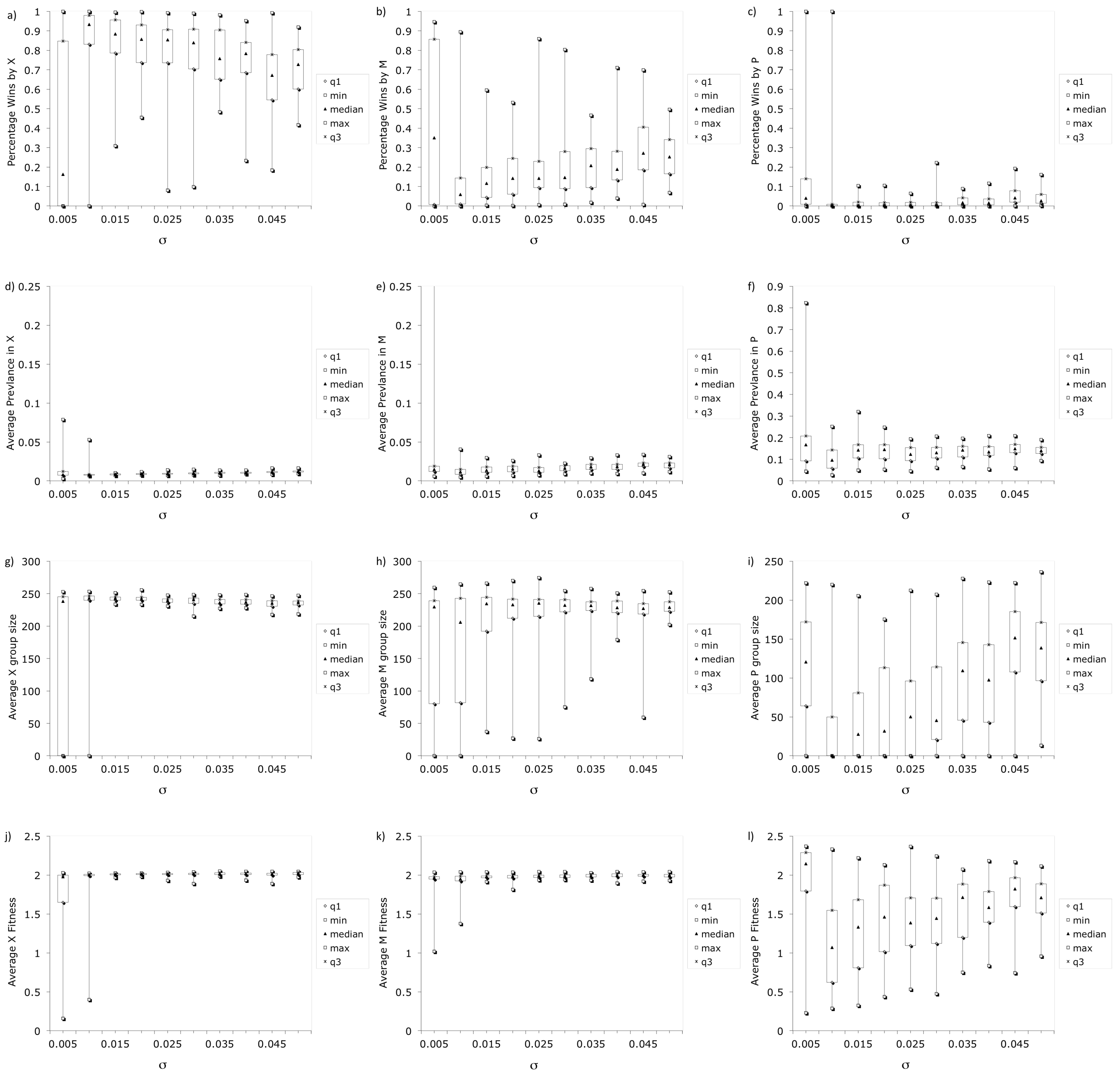

<u>Supplementary Figure 13</u>: Results are insensitive to the proportion of new recruits choosing strategies randomly. Univariate sensitivity analysis with respect to σ, the fraction of newly recruited individuals choosing a strategy randomly, for: percentage of groups at end of simulation dominated by X, M and P individuals **(a-c)**; average infection prevalence in X, M and P individuals **(d-f)**; average size of groups dominated by X, M and P individuals **(g-i)**; and average fitness of X, M and P individuals **(j-k)**. 'X' denotes punishing monogamists, 'M' denotes non-punishing monogamists, 'P' denotes polygynists. Boxplots show median (▲), first quartile (◇), third quartile (*), minimal value observed (□) and maximal value observed (□).

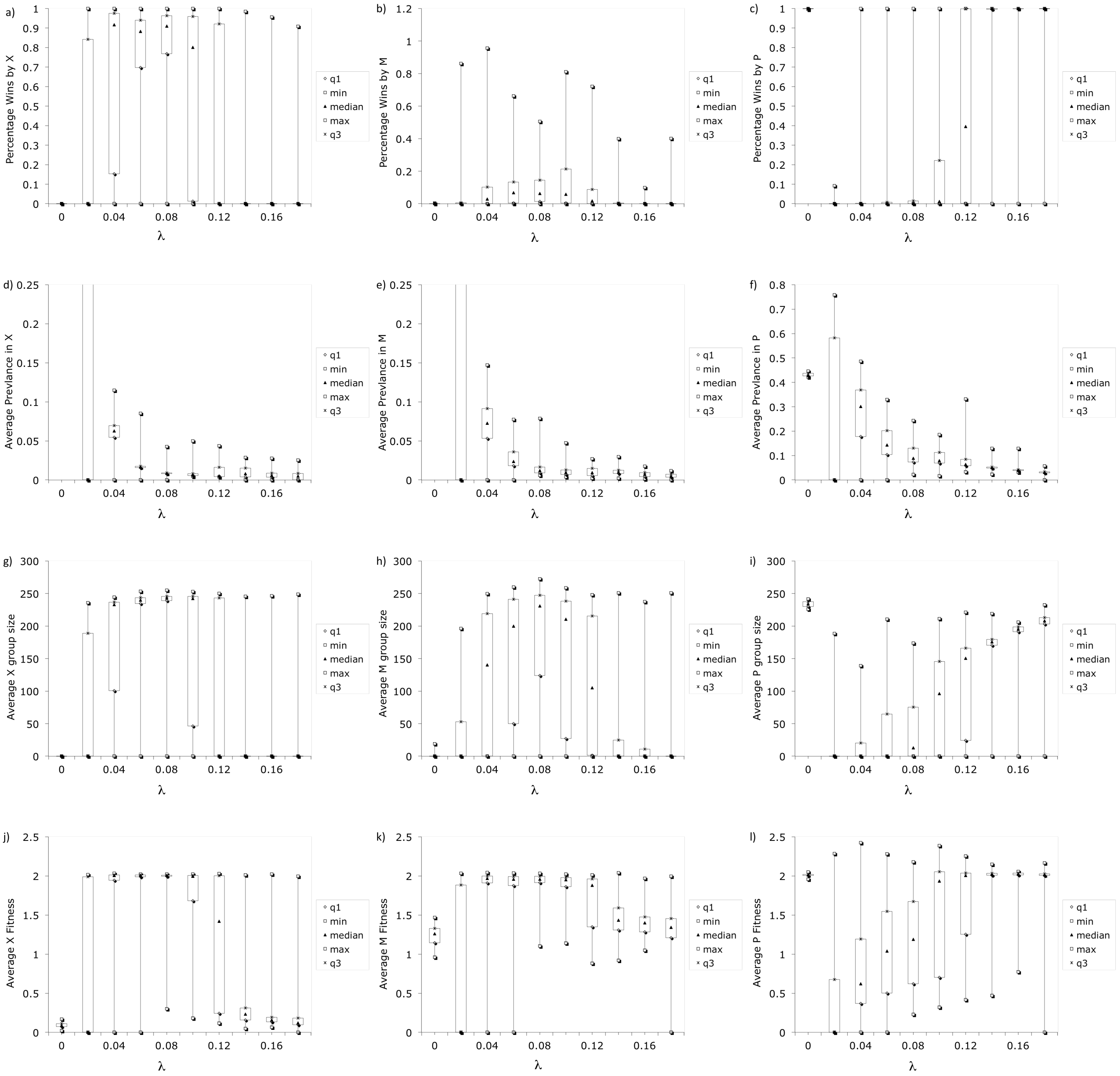

<u>Supplementary Figure 14</u>: A short duration of infection prevents invasion of monogamists by eliminating infection; a long duration suppresses all groups. Univariate sensitivity analysis with respect to λ, the probability per month of clearing infection, for: percentage of groups at end of simulation dominated by X, M and P individuals **(a-c)**; average infection prevalence in X, M and P individuals **(d-f)**; average size of groups dominated by X, M and P individuals **(g-i)**; and average fitness of X, M and P individuals **(j-k)**. 'X' denotes punishing monogamists, 'M' denotes non-punishing monogamists, 'P' denotes polygynists. Boxplots show median (▲), first quartile (◇), third quartile (✱), minimal value observed (□) and maximal value observed (□).

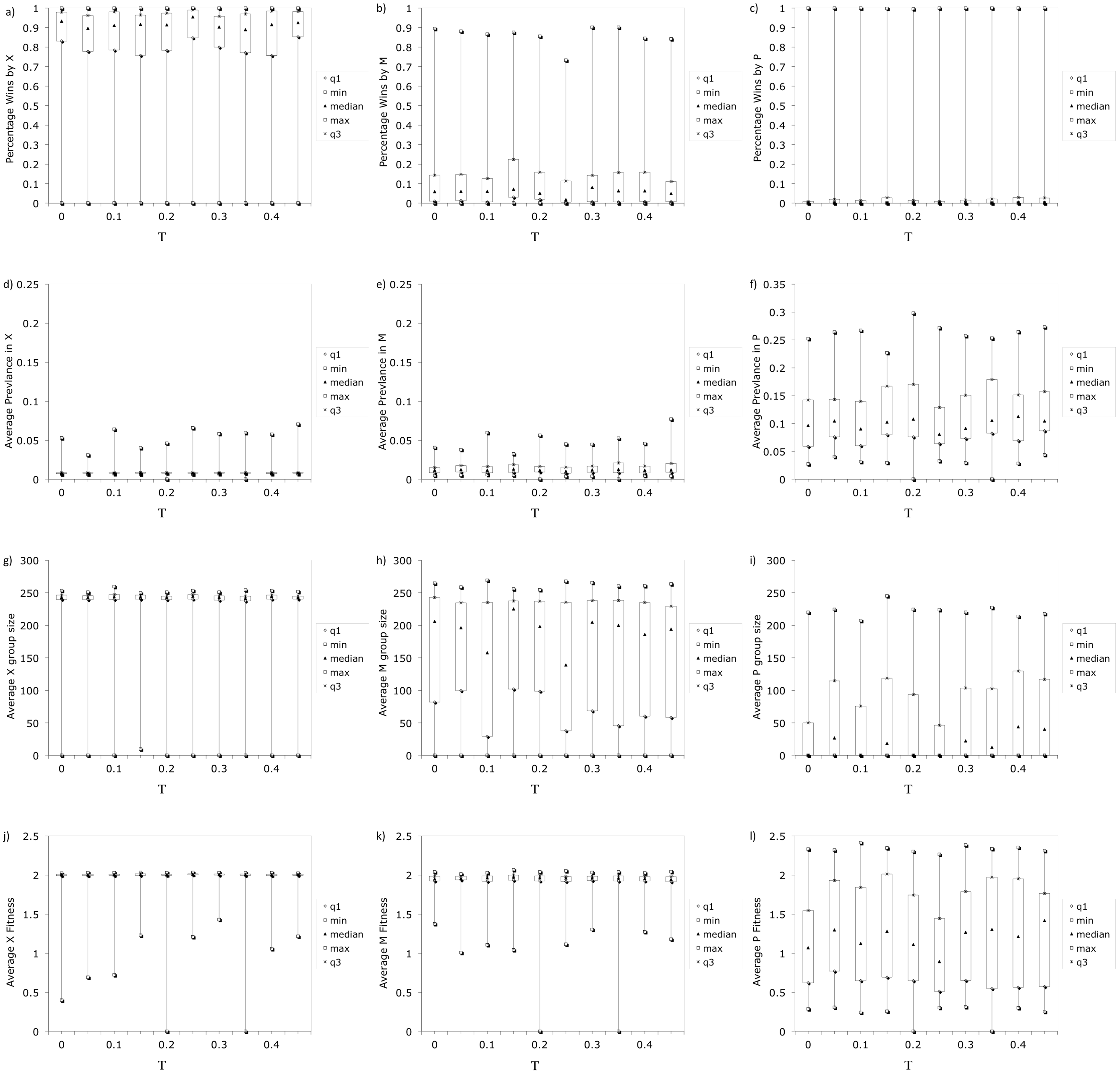

<u>Supplementary Figure 15</u>: Results are insensitive to introducing a threshold density beyond which punishers punish. Univariate sensitivity analysis with respect to T, the density of punishers below which punishers do not punish, for: percentage of groups at end of simulation dominated by X, M and P individuals **(a-c)**; average infection prevalence in X, M and P individuals **(d-f)**; average size of groups dominated by X, M and P individuals **(g-i)**; and average fitness of X, M and P individuals **(j-k)**. 'X' denotes punishing monogamists, 'M' denotes non-punishing monogamists, 'P' denotes polygynists. Boxplots show median (▲), first quartile (◇), third quartile (*), minimal value observed (□) and maximal value observed (□).

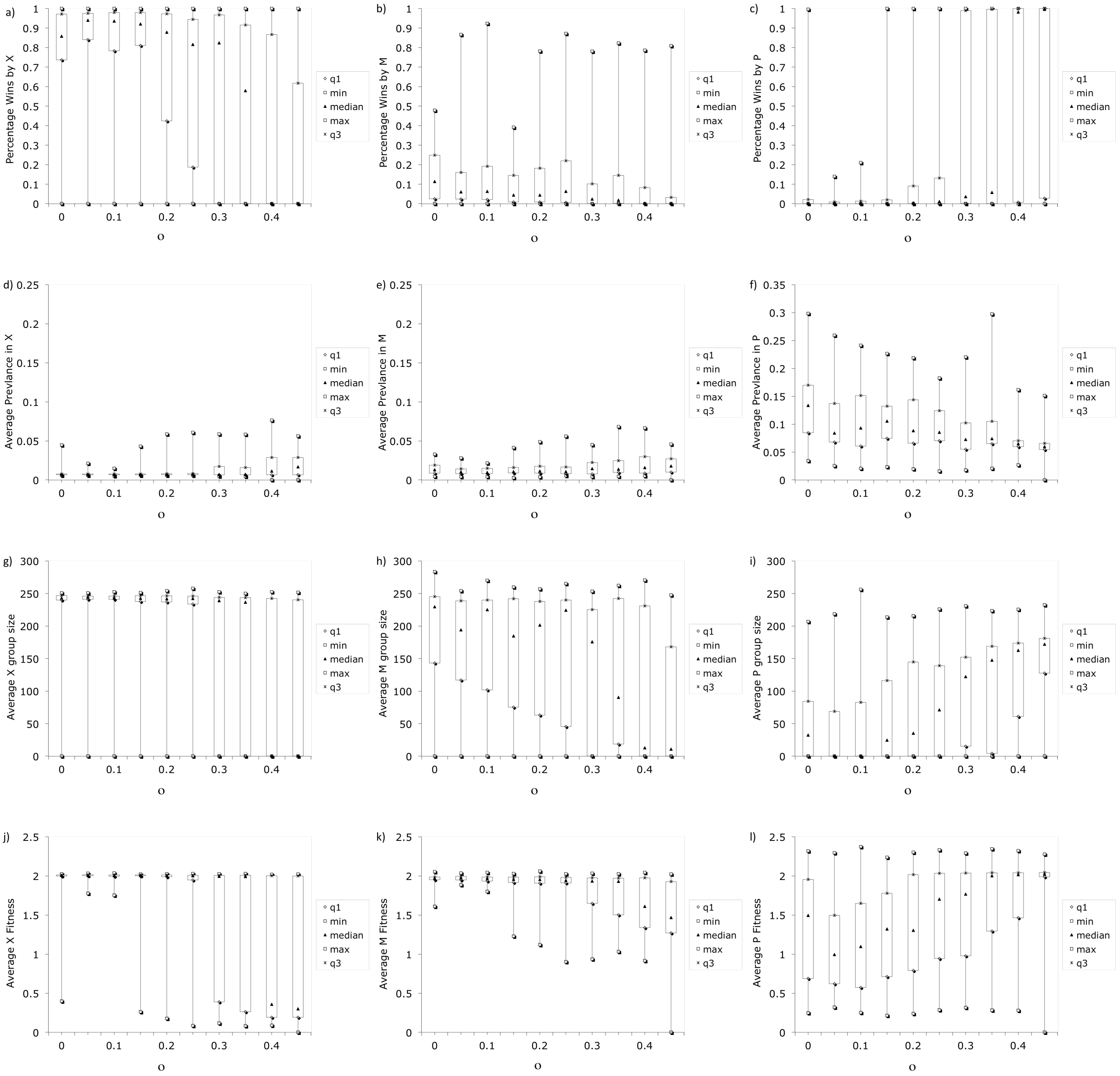

<u>**Supplementary Figure 16**</u>: **Realistic levels of coital dilution do not prevent invasion by punishing monogamists (realistic range: 0.05 < o < 0.25; see Text S1).** Univariate sensitivity analysis with respect to T, the proportion , for: percentage of groups at end of simulation dominated by X, M and P individuals **(a-c)**; average infection prevalence in X, M and P individuals **(d-f)**; average size of groups dominated by X, M and P individuals **(g-i)**; and average fitness of X, M and P individuals **(j-k)**. 'X' denotes punishing monogamists, 'M' denotes non-punishing monogamists, 'P' denotes polygynists. Boxplots show median (▲), first quartile (◇), third quartile (*), minimal value observed (□) and maximal value observed (□).

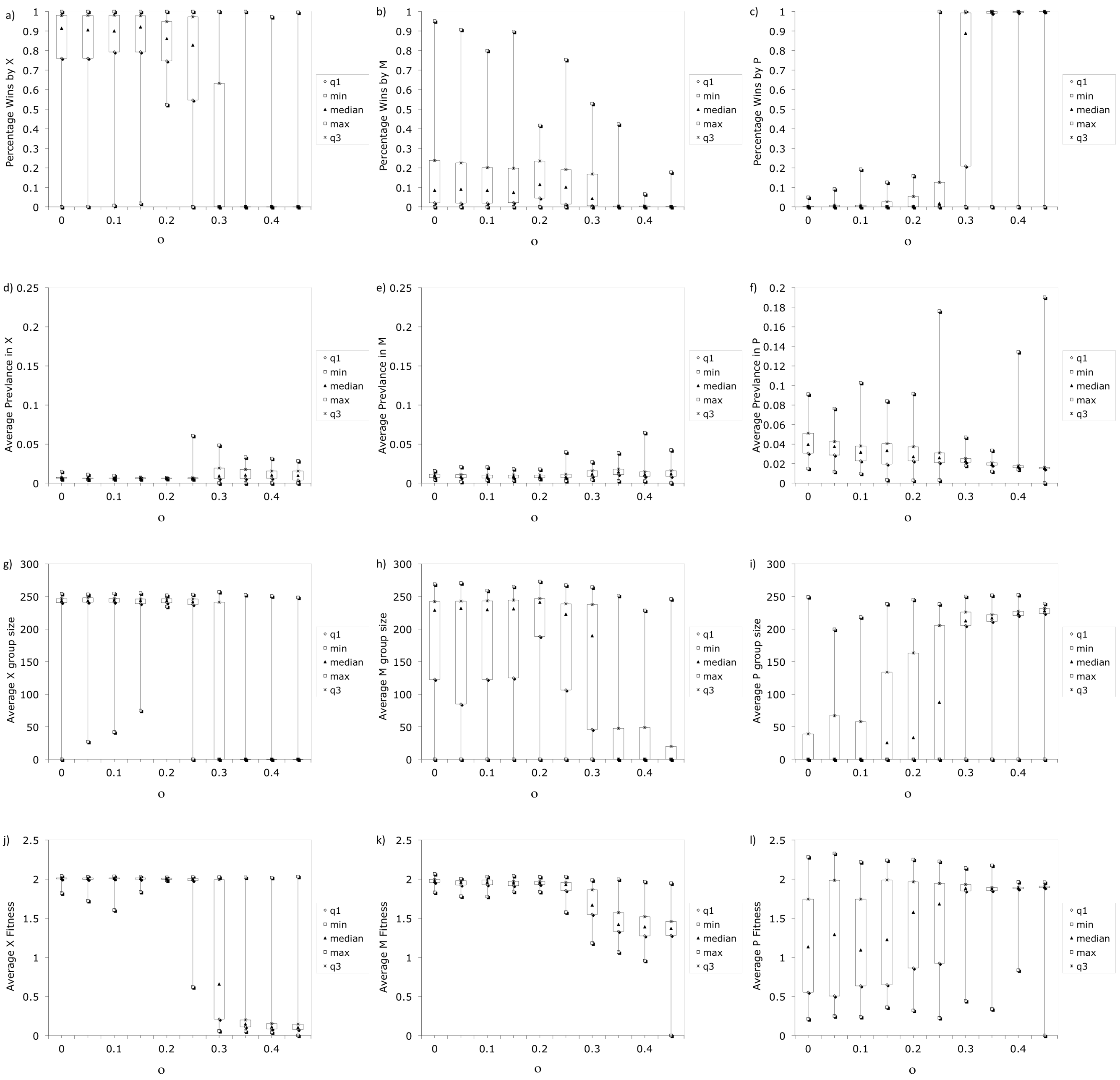

<u>Supplementary Figure 17</u>: Chronic infection scenario: realistic levels of coital dilution prevent invasion by punishing monogamists (realistic range: 0.2 < o < 0.6; see Text S1). Univariate sensitivity analysis with respect to T, the proportion , for: percentage of groups at end of simulation dominated by X, M and P individuals **(a-c)**; average infection prevalence in X, M and P individuals **(d-f)**; average size of groups dominated by X, M and P individuals **(g-i)**; and average fitness of X, M and P individuals **(j-k)**. 'X' denotes punishing monogamists, 'M' denotes non-punishing monogamists, 'P' denotes polygynists. Boxplots show median (▲), first quartile (◇), third quartile (*), minimal value observed (▫) and maximal value observed (▫).

Supplementary Table 1. Data on polygyny and group size. The following data come from the standard cross cultural sample in Murdock and Whites' ethnographic atlas (1) (accessible at http://lucy.ukc.ac.uk/cgi-bin/uncgi/Ethnoatlas/atlas.vopts). Cell entries are the number of groups in each category.

Group size	<50	50-99	100-199	200-399	400-999	1000-4999	5000-49999	50000+	Total
Monogamy	2	2	3	0	3	1	0	6	21
Polygyny (sororal or non-sororal)	12	13	11	12	6	1	4	6	39
Total	14	15	14	12	9	2	4	12	60

Supplementary Table 2. STI prevalence and group size by strategy for chronic infection scenario. Long-term average group sizes and STI prevalence by strategy type, within monogamist (X), non-punishing monogamist (M), and polygynists (P) groups and across the whole population, for the small group and large group scenarios. Values are the average across 100 simulation runs; parenthetical values denote one standard deviation.

		Small group scenario				Large group scenario			
		Within X groups	Within M groups	Within P groups	Across all groups	Within X groups	Within M groups	Within P groups	Across all groups
STI prevalence	All individuals	0.009 (±0.004)	0.008 (±0.003)	0.007 (±0.0002)	0.007 (±0.0002)	0.01 (±0.001)	0.01 (±0.002)	0.04 (±0.01)	0.01 (±0.002)
	X individuals	0.008 (±0.003)	0.005 (±0.009)	0.004 (±0.001)	0.005 (±0.001)	0.01 (±0.001)	0.01 (±0.001)	0.03 (±0.01)	0.01 (±0.001)
	M individuals	0.006 (±0.017)	0.007 (±0.003)	0.005 (±0.001)	0.005 (±0.001)	0.01 (±0.0003)	0.01 (±0.0003)	0.03 (±0.004)	0.01 (±0.001)
	P individuals	0.017 (±0.008)	0.015 (±0.007)	0.007 (±0.0002)	0.007 (±0.0002)	0.03 (±0.002)	0.05 (±0.004)	0.05 (±0.01)	0.04 (±0.004)
Group Size		20.6 (±0.8)	20.8 (±0.7)	21.7 (±0.1)	21.6 (±0.1)	243.3 (±1.1)	240.6 (±4.4)	223.1 (±9.8)	239.4 (±4.4)

Supplementary Methods

Bacterial STI scenario. Partnering rates are high in hunter-gatherer (forager) groups (except in polygynous societies among men who cannot find mates) (2), hence we choose ρ=0.1/month for the baseline probability per month that a single female seeks a partner. Exogamy rates are similarly high (3), hence we choose ε=0.80 for the baseline probability that a single female who is looking for a partner, looks outside their current group. Divorce rates vary depending on the number of years partners have been together, however, the average dissolution rate over the long term is about 5% per year (4), hence we choose the pair breakup probability of ω=0.05/year.

The normal human lifespan for a healthy hunter-gatherer is about 70 years (5), hence we chose δ=0.01429/year for the probability per year that an individual dies due to causes other than sexually transmitted infections. The total fertility rate (births per lifetime) among female hunter-gatherers is about 5.5 with a standard deviation of 1.69 (6). For a lognormal distribution, these values correspond to a lognormal location parameter of μ=-5.03 and a lognormal scale parameter of s=0.29. This corresponds to a mean birth probability per unit time of β=0.079/year.

The mate selection exponent was chosen by calibrating the model to match data on the distribution of the number of female partners per male in polygynous societies (7), yielding χ=1.5. In hunter-gatherers, men provide for at least 40% of the diet, although societies are variable and there are many cases where they provide more than 50% (6, 8). Hence, we assumed ϕ=0.3, corresponding to males providing 30% of the diet. This is a conservative estimate since it tends to favour polygyny, by increasing the birth rate in polygynous partnerships. Lower values than ϕ=0.3 were avoided because lower values resulted in highly skewed partnership distributions that did not match empirical data (7).

We assumed that a fraction σ=0.01 of newly recruited individuals picked a strategy randomly rather than picking a successful strategy. We assumed that the infection was re-introduced from other metapopulations (e.g. through travel) every Δ=70 years, in a proportion π=0.01 of randomly chosen individuals.

The reported transmission rates for bacterial STIs such as chlamydia, gonorrhea and syphilis are variable, but generally very high, with a transmission probability of approximately 10-50% per sexual contact (9-12). Assuming S=8 sex acts per month per partnership with a probability of transmission P=0.2 per sex act, we find from the equation $\tau = 1 - (1 - P)^S$ that the probability that infection is transmitted from an infected partner to a susceptible partner is τ=0.85/month.

The reported duration of untreated infection is also variable and it is difficult to acquire data on, since bacterial STI infections are usually treated (13, 14). For chlamydia, the duration of untreated infection has been estimated to be 1 year on average (15), although for some the duration can be up to 2 years (16). Gonorrhea appears to have a shorter duration of infection than chlamydia (13, 14). The primary and secondary stages of syphilis are about 6 months (17) but infection is lifelong, relapse to the secondary phase is

very common, and the early latent phase may be infectious (18). Based on these studies we choose a baseline value of λ=1.0/year for the probability of clearing infection (and becoming fully susceptible again).

Infertility rates due to bacterial STI infections vary among populations. For instance, the rate of pelvic inflammatory disease (PID) among women with unscreened chlamydia infection higher income populations is about 10%, and PID is estimated to cause infertility in about 16% of cases (19, 20). However, STI-caused infertility rates appear to be higher in lower income populations with limited access to medical interventions (21). We choose a relatively low value of θ=0.05 for the probability that an STI causes infertility.

We assumed that punishing monogamists pay a cost c=0.01 to punish each polygynist in their group, and that polygynists receive the same penalty c=0.01 for punishing monogamist in their group. For a punishing monogamist group of size 250 (with approximately 125 males), c=0.01 means that polygynists receive the maximum possible punishment due to the aggregate effects from all the punishing monogamists in the group.

The group sizes for the small group (hunter-gatherer) and large group (agriculturalist) scenarios were taken from empirical data on group sizes (6). We assumed a probability κ=0.05/month that two groups in the metapopulation compete (significantly larger values made the metapopulation unsustainable due to excessive population reduction), and a controlling factor γ=10 corresponds to moderate effects of group size in the outcomes of group conflicts.

Chronic infection scenario. For the chronic infection scenario, we based our parameters on Human Immunodeficiency Virus (HIV) infections. We assumed S=8 sex acts per month, and a per act transmission probability of P=0.005 based on data in low-income populations (22), hence we find from the equation $\tau = 1 - (1-P)^S$ that the probability that infection is transmitted from an infected partner to a susceptible partner is τ=0.04/month. The duration of infection was assumed to be lifelong, but the duration of life while infected was assumed to be 10 years, hence a death rate of 0.1/year due to STI infection (23). Infertility rates are high in HIV infection (24) hence we assumed θ=0.5.

Coital dilution scenarios. To capture coital dilution in our sensitivity analysis we assumed that coital dilution reduces the per-month transmission probability by a multiplicative factor

$$\frac{1}{1-o+oN} \quad (1)$$

where N is the number of partners, and o controls the impact of coital dilution. The factor o can be computed from the equation

$$o = \frac{1}{N-1}\left[\frac{1-(1-P)^S}{1-(1-P)^{S\times OR}} - 1\right] \quad (2)$$

where P is the per act transmission rate, S is the baseline number of sex acts per month, and OR is the odds ratio for the reduced frequency of sex acts in situations where there are multiple partners, which can be taken from empirical data (25-27). This equation was in

turn derived from the equation relating per-month and per-act transmission probabilities $\tau = 1 - (1 - P)^S$. From an empirical study on coital dilution in polygynous relationships (26), the odds ratio OR=0.84 for 2 partners, and OR=0.44 for 3 or more partners. As before, S=8 acts per months. For bacterial STIs with τ=0.85/month, this yields $o \in [0.07, 0.26]$. For HIV with τ=0.04/month, this yields $o \in [0.19, 0.62]$. This approach is conservative, since it does not account for extramarital sex in partnerships where females experience coital dilution to the presence of other partners to their mate.

Supplementary References

1. Murdock GP & White DR (1969) Standard cross-cultural sample. *Ethnology* 8(4):329-369.
2. Marlowe FW (2003) The mating system of foragers in the standard cross-cultural sample. *Cross-Cultural Research* 37(3):282-306.
3. Fix AG (1999) *Migration and colonization in human microevolution* (Cambridge University Press).
4. Blurton Jones NG, Marlowe FW, Hawkes K, & O'Connell JF (2000) Paternal investment and hunter-gatherer divorce rates. *Adaptation and human behavior: An anthropological perspective, ed. L. Cronk, N. Chagnon & W. Irons*:69-90.
5. Gurven M & Kaplan H (2007) Longevity among hunter-gatherers: a cross-cultural examination. *Population and Development Review* 33(2):321-365.
6. Marlowe FW (2005) Hunter-gatherers and human evolution. *Evolutionary Anthropology: Issues, News, and Reviews* 14(2):54-67.
7. White AA (2013) Subsistence economics, family size, and the emergence of social complexity in hunter–gatherer systems in eastern North America. *Journal of Anthropological Archaeology* 32(1):122-163.
8. Codding BF, Bird RB, & Bird DW (2011) Provisioning offspring and others: risk–energy trade-offs and gender differences in hunter–gatherer foraging strategies. *Proceedings of the Royal Society B: Biological Sciences* 278(1717):2502-2509.
9. LYCKE E, LOWHAGEN G-B, HALLHAGEN G, JOHANNISSON G, & RAMSTEDT K (1980) The risk of transmission of genital Chlamydia trachomatis infection is less than that of genital Neisseria gonorrhoeae infection. *Sexually transmitted diseases* 7(1):6-10.
10. Kretzschmar M, van Duynhoven YT, & Severijnen AJ (1996) Modeling prevention strategies for gonorrhea and chlamydia using stochastic network simulations. *American Journal of Epidemiology* 144(3):306-317.
11. Kretzschmar M, Welte R, Van den Hoek A, & Postma MJ (2001) Comparative model-based analysis of screening programs for Chlamydia trachomatis infections. *American journal of epidemiology* 153(1):90-101.
12. POTTERAT JJ, DUKES RL, & ROTHENBERG RB (1987) Disease transmission by heterosexual men with gonorrhea: an empiric estimate. *Sexually transmitted diseases* 14(2):107-110.
13. WHO (2011) Prevalence and incidence of sexually transmitted infections. http://whqlibdoc.who.int/publications/2011/9789241502450_eng.pdf.
14. Golden MR, Schillinger JA, Markowitz L, & Louis MES (2000) Duration of Untreated Genital Infections WithChlamydia trachomatis: A Review of the Literature. *Sexually transmitted diseases* 27(6):329-337.

15. Buhaug H, Skjeldestad FE, Backe B, & Dalen A (1989) Cost effectiveness of testing for chlamydial infections in asymptomatic women. *Medical care*:833-841.
16. Rahm V, Belsheim J, Gleerup A, Gnarpe H, & Rosen G (1986) Asymptomatic carriage of Chlamydia-trachomatis-a study of 109 teenage girls. *European Journal of Sexually Transmitted Diseases* 3(2):91-94.
17. Garnett GP, Aral SO, Hoyle DV, Cates Jr W, & Anderson RM (1997) The natural history of syphilis: implications for the transmission dynamics and control of infection. *Sexually transmitted diseases* 24(4):185-200.
18. Evans AS (1991) *Bacterial infections in humans: epidemiology and control* (Plenum, New York).
19. Oakeshott P*, et al.* (2010) Randomised controlled trial of screening for Chlamydia trachomatis to prevent pelvic inflammatory disease: the POPI (prevention of pelvic infection) trial. *Bmj* 340:c1642.
20. Phillips AJ (2006) Chlamydial infections. *Sexually Transmitted Diseases*, (Springer), pp 127-151.
21. Caldwell JC & Caldwell P (1983) The demographic evidence for the incidence and cause of abnormally low fertility in tropical Africa. *World health statistics quarterly. Rapport trimestriel de statistiques sanitaires mondiales* 36(1):2-34.
22. Boily M-C*, et al.* (2009) Heterosexual risk of HIV-1 infection per sexual act: systematic review and meta-analysis of observational studies. *The Lancet infectious diseases* 9(2):118-129.
23. Jaffar S, Grant AD, Whitworth J, Smith PG, & Whittle H (2004) The natural history of HIV-1 and HIV-2 infections in adults in Africa: a literature review. *Bulletin of the World Health Organization* 82(6):462-469.
24. Gray RH*, et al.* (1998) Population-based study of fertility in women with HIV-1 infection in Uganda. *The lancet* 351(9096):98-103.
25. Gaydosh L, Reniers G, & Helleringer S (2013) Partnership concurrency and coital frequency. *AIDS and behavior* 17(7):2376-2386.
26. Stewart H, Morison L, & White R (2002) Determinants of coital frequency among married women in Central African Republic: the role of female genital cutting. *Journal of Biosocial Science* 34(04):525-539.
27. Reniers G & Tfaily R (2012) Polygyny, partnership concurrency, and HIV transmission in sub-Saharan Africa. *Demography* 49(3):1075-1101.
